# A Dual‐Purpose Non‐Canonical Amino Acid for the Expanded Genetic Code: Combining Metal‐Binding and Click Chemistry

**DOI:** 10.1002/anie.202413073

**Published:** 2024-10-31

**Authors:** Graham J. Day, Andrey V. Zaytsev, Richard C. Brewster, Valery N. Kozhevnikov, Amanda G. Jarvis

**Affiliations:** ^1^ EaStCHEM School of Chemistry University of Edinburgh Joseph Black Building EH9 3FJ Edinburgh UK; ^2^ Department of Applied Sciences Northumbria University NE1 8ST Newcastle-upon-Tyne UK

**Keywords:** genetic code expansion, non-canonical amino acids, iridium, bioorthogonal reactivity, luminescent probes

## Abstract

A rationally designed dual‐purpose non‐canonical amino acid (Trz) has been synthesised and successfully incorporated into a protein scaffold by genetic code expansion. Trz contains a 5‐pyridyl‐1,2,4‐triazine system, which allows for inverse‐electron‐demand Diels–Alder (IEDDA) reactions to occur on the triazine ring and for metal ions to be chelated both before and after the click reaction. Trz was successfully incorporated into a protein scaffold and the IEDDA utility of Trz demonstrated through the site‐specific labelling of the purified protein with a bicyclononyne. Additionally, Trz was shown to successfully coordinate a cyclometallated iridium(III) centre, providing access to a bioorthogonal luminogenic probe. The luminescent properties of the Ir(III)‐bound protein blue‐shift upon IEDDA click reaction with bicyclononyne, providing a unique method for monitoring the extent and location of the labelling reaction. In summary, Trz is a new dual‐purpose non‐canonical amino acid with great potential for myriad bioapplications where metal‐based functionality is required, for example in imaging, catalysis, and photo‐dynamic therapy, in conjunction with a bioorthogonal reactive handle to impart additional functionalities, such as dual‐modality imaging or therapeutic payloads.

## Graphical abstract

Genetic code expansion strategies tend to incorporate non‐canonical amino acids (ncAAs) with singular, specialised functions into proteins. In this study, a dual‐purpose ncAA that can undergo inverse‐electron‐demand Diels–Alder (IEEDA) reactions and coordinate luminogenic metal complexes was designed, synthesised, and incorporated into a protein. Significantly, luminescence studies revealed emission shifts upon dual functionalisation.

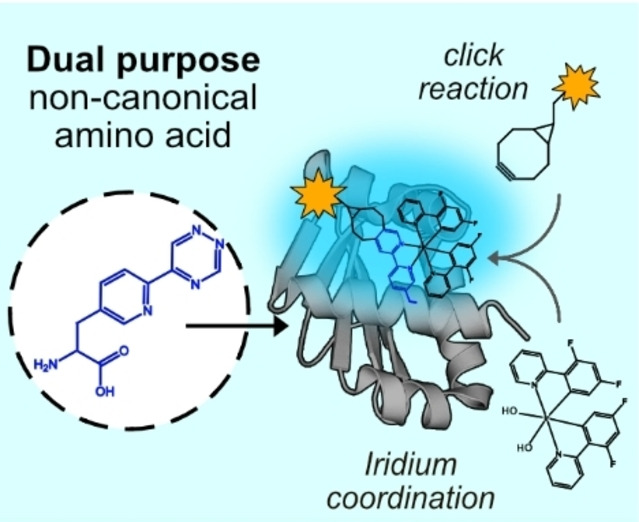

Site‐selective protein functionalisation allows the introduction of desired properties at a defined site within a protein structure. The resulting proteins are of interest for a range of studies from those mimicking post‐translational modifications to understand more about their biological functions,^[1]^ to the design of completely new‐to‐nature active sites in enzymes.^[2]^ Whilst natural proteins can be modified through the presence of reactive amino acid side chains, for example cysteines, the presence of multiple of the same residues within a protein scaffold limits this approach as a truly selective method to obtain protein conjugates. The expanded genetic code provides an alternative method to access precisely targeted protein conjugates through the incorporation of non‐canonical amino acids (ncAA).^[3]^ Using this methodology, ncAAs for multiple applications have been introduced to proteins and include post‐translational modification mimics,[^4^, ^5^, ^6^, ^7^] metal‐binding amino acids,[^8^, ^9^, ^10^] catalytic amino acids,[^11^, ^12^, ^13^, ^14^] and ncAAs with bioorthogonal reactive handles.

The majority of examples of genetic code expansion (GCE) for protein modification have focused on generating bioconjugates with a single functionality (Figure 1; top). For several applications, it is desirable to access dual‐modified protein bioconjugates that allow the use of two different ncAAs providing different functionalities. For example, combining both an imaging agent and therapeutic payload into protein‐based drugs for precision medicine (theranostics).^[15]^ Dual and multiply modified proteins can be accessed by GCE methods, but they are often unwieldy and low yielding, requiring the different orthogonal translation systems for each amino acid to act together. The orthogonal aaRSs must have good relative fidelity for each amino acid, exhibit minimal polyspecificity, must not cross acylate the different tRNAs, and must only recognise the desired codon (i.e. amber and ochre).^[16]^ Often, this requires extensive optimisation and engineering. Bifunctional ncAAs containing multiple different reactive handles, such as a SPAAC and an IEDDA site, offer one way to reduce the challenges with dual functionalisation whilst maintaining the ability to introduce multiple functionalities into a protein.[^17^, ^18^, ^19^, ^20^] However, no bifunctional ncAA containing a metal binding site has been incorporated into the genetic code.

**Figure 1:**
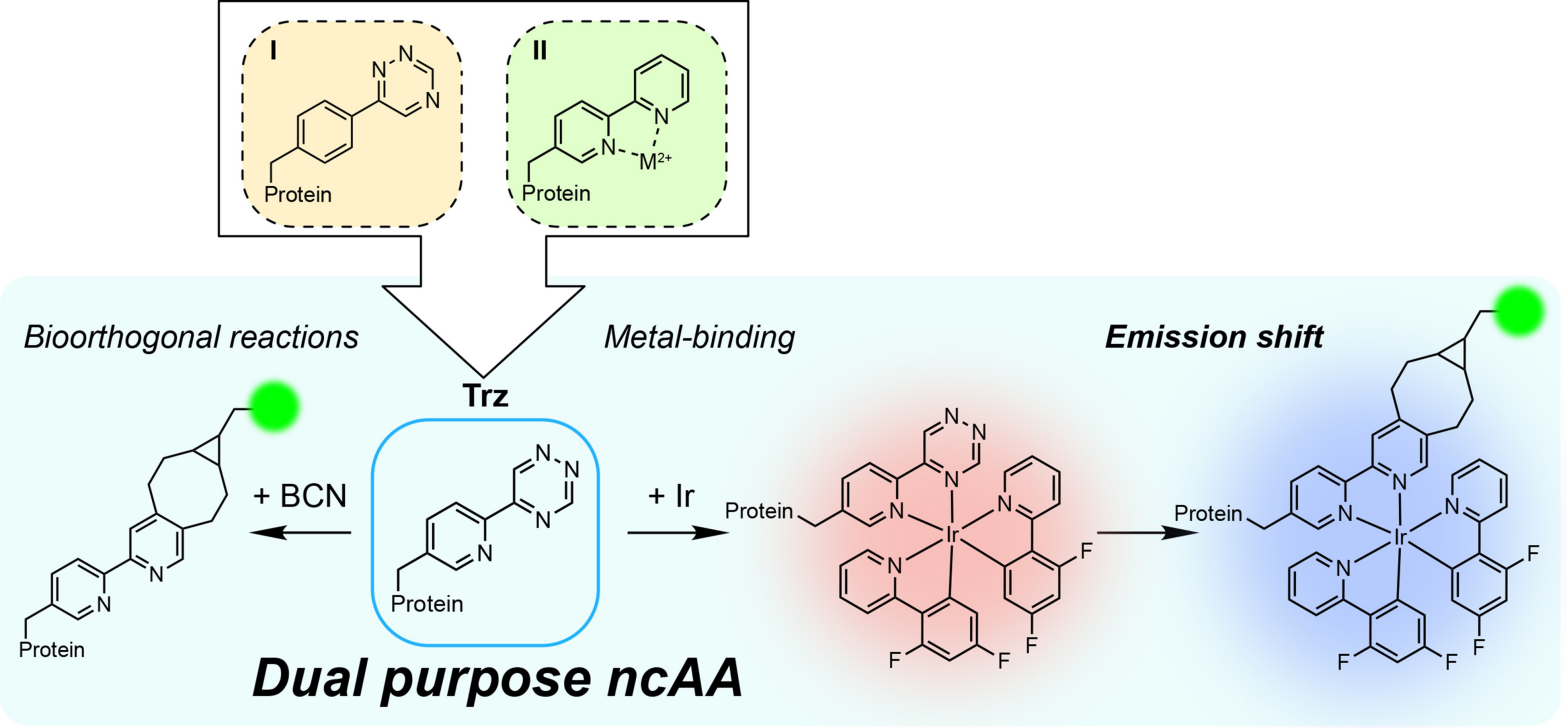
Top: Previously reported non‐canonical amino acids (ncAAs) containing a 1,2,4‐triazine (**I**) or 2,2’‐bipyridine (**II**) moiety, for bioorthogonal reactions or metal binding, respectively. Mx^2+^ ion could be Ni^2+^, Cu^2+^, Co^2+^, Fe^2+^, or Zn^2+^. Bottom: The new ncAA Trz presented herein, which can undergo bioorthogonal reactions (left) and can coordintate a cyclometallated iridium(III) centre. Furthermore, Trz exhibits dual functionality in that it can facilitate both of these different applications successively, providing a route to dual‐functional site‐specific protein labelling applications. The green circle represents a possible functional group, such as a fluorescent reporter, coupled to the BCN.

Herein, we report the design of a dual‐purpose non‐canonical amino acid, 5‐(pyridin‐2‐yl)‐1,2,4‐triazine alanine, Trz, containing a 1,2,4‐triazine to be utilised in bioorthogonal labelling reactions and a *N*,*N*‐chelator to allow formation of protein–metal conjugates (Figure 1;). For the bioorthogonal reactive handle we choose a triazine as they undergo IEDDA reactions, the fastest bioorthogonal reactions known to date,[^21^, ^22^, ^23^, ^24^, ^25^, ^26^, ^27^] and have been suggested to exhibit greater stability under physiological conditions than their tetrazine equivalents, albeit with significant loss in reactivity.[^24^, ^25^] Triazines alone undergo the IEDDA reactions at rates below the minimum required for effective bioorthogonal reactions (1 M^−1^ ⋅ s^−1^).^[28]^ Therefore a pyridyl group was added to the 5 position of 1,2,4‐triazine to form a bidentate *N*,*N‐*chelator for metal coordination as previous work on 5‐(pyridin‐2‐yl)‐1,2,4‐triazine established that the chelation of Ir^III^ catalyses the IEDDA reaction with BCN−OH, accelerating the rate constant over 130‐fold from 0.059 to 7.78 M^−1^ ⋅ s^−1^.^[27]^ An increase in IEDDA reaction rate was also observed with Re^I^ albeit it to a lesser extent (55‐fold).^[29]^ The resulting iridium(III) complexes can also act as luminescent probes, exhibiting favourable properties for bioimaging, including enhanced photostabilities over organic dyes, increased resolution through less self‐quenching effects, deeper tissue penetration, and compatibility with time‐resolved imaging methods to avoid background autofluorescence.[^30^, ^31^] Previous work has shown that the luminescent properties of the iridium complex change on BCN addition allowing the success of the reaction to be monitored via luminescence.^[27]^ Moreover, the ability to post‐functionalise a probe to shift its luminescent properties is an attractive feature, providing a switch‐on reporter for in vivo or in vitro protein–protein interaction studies^[32]^ or monitoring endogenously expressed proteins.^[33]^

The ncAA Trz was synthesised as depicted in Scheme 1. The intermediate 5‐(5‐methylpyridin‐2‐yl)‐1,2,4‐triazine (**3**) was initially synthesised, via the oxidation of 2‐acetyl‐5‐methylpyridine (**1**) to the corresponding glyoxal derivative followed by condensation with thiosemicarbazide to form 3‐mercapto‐5(methylpyridine‐2‐yl)‐1,2,4‐triazine. Finally, nucleophilic aromatic substitution of the thiol with hydrazine to give **2**, followed by dehydrazination under strongly basic conditions gave **3**.^[27]^ Free radical bromination of **3** led to the bromomethyl compound (**4**), which was used to alkylate diethyl (Boc‐amino)malonate in the presence of sodium hydride as a base, giving compound **5**. Treatment of the ester using 2.2 molar equivalents of 1 m aqueous LiOH at room temperature for 20 hours followed by 2 m HCl led to incomplete hydrolysis giving the ethyl ester of Trz. Increasing the amount of 1 m LiOH to 4.4 equivalents followed by heating under reflux with 2 m HCl led to the desired ncAA Trz.

**Scheme 1:**
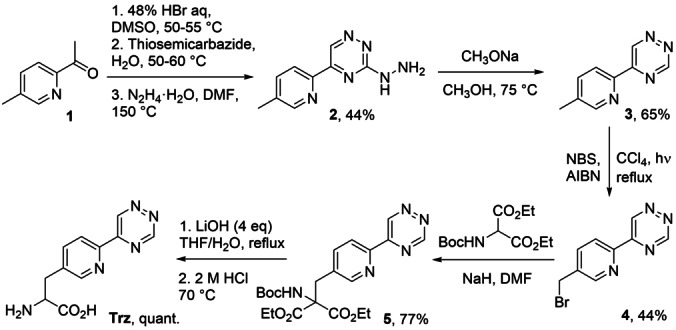
Synthetic route to Trz. NBS=*N‐*bromosuccinimide, AIBN=azobisisobutyronitrile. For full details, please refer to the Supporting Information.

Trz is structurally similar to bipyridylalanine (BpyAla; Figure 1), which has been incorporated into multiple proteins using an evolved orthogonal tRNA/aaRS from *Methanococcus jannaschii*.^[8]^ Computational docking of Trz into the bipyridylalanine tRNA synthetase (pdb: 2PXH) indicated that Trz would be accepted by the *Mj*TyrRS(ByRS)/*Mj*tRNA^Tyr^ pair and that incorporation into a protein scaffold using this existing translation system would be viable (Figure 2a). The sterol carrier protein (SCP‐2 L) from the human multifunctional enzyme 2 was chosen as a model protein scaffold due to its ease of production and extensive use in artificial enzyme design,[^34^, ^35^, ^36^, ^37^] including the incorporation of BpyAla to bind catalytic copper ions.^[38]^ The TAG stop codon was introduced into the SCP gene at M112, a surface position (Figure 2b), to increase the availability of the 1,2,4‐triazine for the IEDDA reactions. *Escherichia coli* cells containing the pEVOL‐BpyAla plasmid expressing the orthogonal *Mj*TyrRS/*Mj*tRNA^Tyr^ pair for ncAA incorporation were transformed with a pET28 plasmid containing a codon optimised gene encoding for SCP_M112TAG with a C‐terminal cleavable polyhistidine (His_6_) tag. Initial expression studies were carried out with the ethyl ester derivatives of Trz and BpyAla (Trz‐Et and BpyAla‐Et, respectively; Figure S19), as the ethyl ester was expected to be cleaved in vivo to produce the amino acid,^[38]^ alongside BpyAla as an uninhibited ncAA control. The production of full‐length protein (15.4 kDa) was verified by the presence of protein bands on SDS–PAGE gels, indicating that in vivo hydrolysis of the ethyl ester to the amino acid had occurred prior to translation (Figure S19). Whilst both amino acid esters were cleaved and incorporated, use of the free ncAA BpyAla gave rise to higher expression than either BpyAla‐Et, or Trz‐Et, presumably due to the higher effective concentration in vivo as no hydrolysis needs to occur. Quantification of products from larger scale expression studies revealed that the yield with BpyAla was twice as high as BpyAla‐Et (6.63 vs 3.30 mg ⋅ L^−1^). Importantly, both Trz‐Et and BpyAla‐Et gave similar quantities of protein, implying that the extra nitrogen atoms on Trz did not significantly hinder incorporation and the ncAA was recognised by the synthetase.

**Figure 2:**
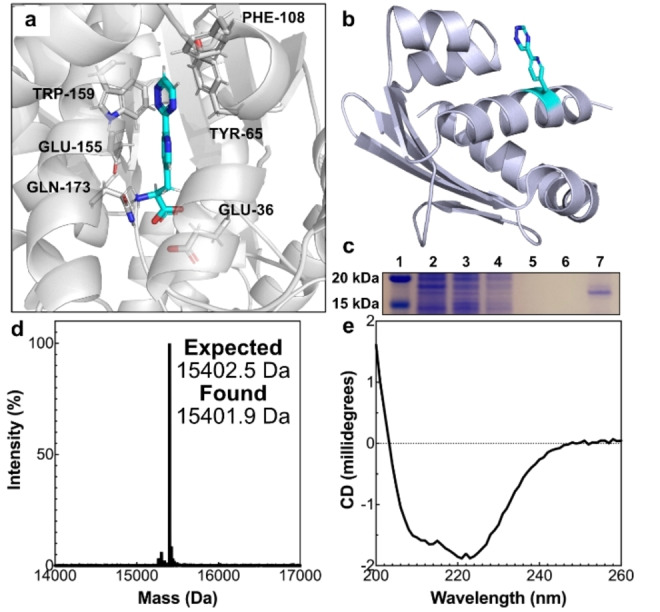
Incorporation of Trz into SCP. a) Results from docking Trz (cyan) into the amino‐acyl tRNA synthetase (grey) from *Methanococcus jannaschii* (PDB: 2PXH). Amino acids that interact with Trz are indicated. b) Schematic representation of Trz presented on the surface of SCP. c) SDS–PAGE gel of purified SCP−His_6__Trz isolated by immobilised metal ion affinity chromatography (IMAC). Lanes: molecular weights standard (1), cell lysate (2), IMAC flow‐through (3), IMAC wash fraction (4), IMAC elution fraction (5), dialysed elution fraction (6), and concentrated dialysed fraction (7). See Figure S20 for full SDS–PAGE gel. d) Deconvoluted mass spectrum of SCP−His_6__Trz revealing a single peak at the expected molecular weight. e) Far‐UV circular dichroism spectra of SCP−His_6__Trz. Samples measured in 30 mM MES buffer, pH 6.0 at 20 °C.

Following these initial studies, SCP_M112TAG was expressed in the presence of Trz, and successfully purified (Figure 2c) to give SCP−His_6__Trz. Liquid chromatography–mass spectrometry (LC–MS) confirmed the identity of the isolated protein as SCP−His_6__Trz, giving a mass of 15401.9 Da (Figure 2d) in line with the predicted mass of SCP−His_6__Trz (15402.5 Da; Supporting Information Table 3). Far‐UV circular dichroism spectroscopy measurements of SCP−His_6__Trz revealed the isolated protein exhibited a spectrum with negative peaks at 222 and 208 nm, indicative of a folded protein displaying α‐helices (Figure 2e).

Following the successful expression of SCP−His_6__Trz, the availability of the 1,2,4‐triazine to participate in an IEDDA reaction was investigated (reaction A; Scheme 2). Incubating 10 μM SCP−His_6__Trz with 10 equivalents of bicyclo[6.1.0]non‐4‐yn‐9‐ylmethanol (BCN−OH) over 18 h gave quantitative conversion to the expected product, SCP−His_6__Trz−BCN−OH (15524.5 Da). Analysis by LC–MS revealed a peak of 15524.4 Da (Figure 3a) matching the expected product mass following BCN−OH addition (+150.2 Da) and loss of dinitrogen (−28.0 Da) to the SCP−His_6__Trz (15401.9 Da).

**Scheme 2:**
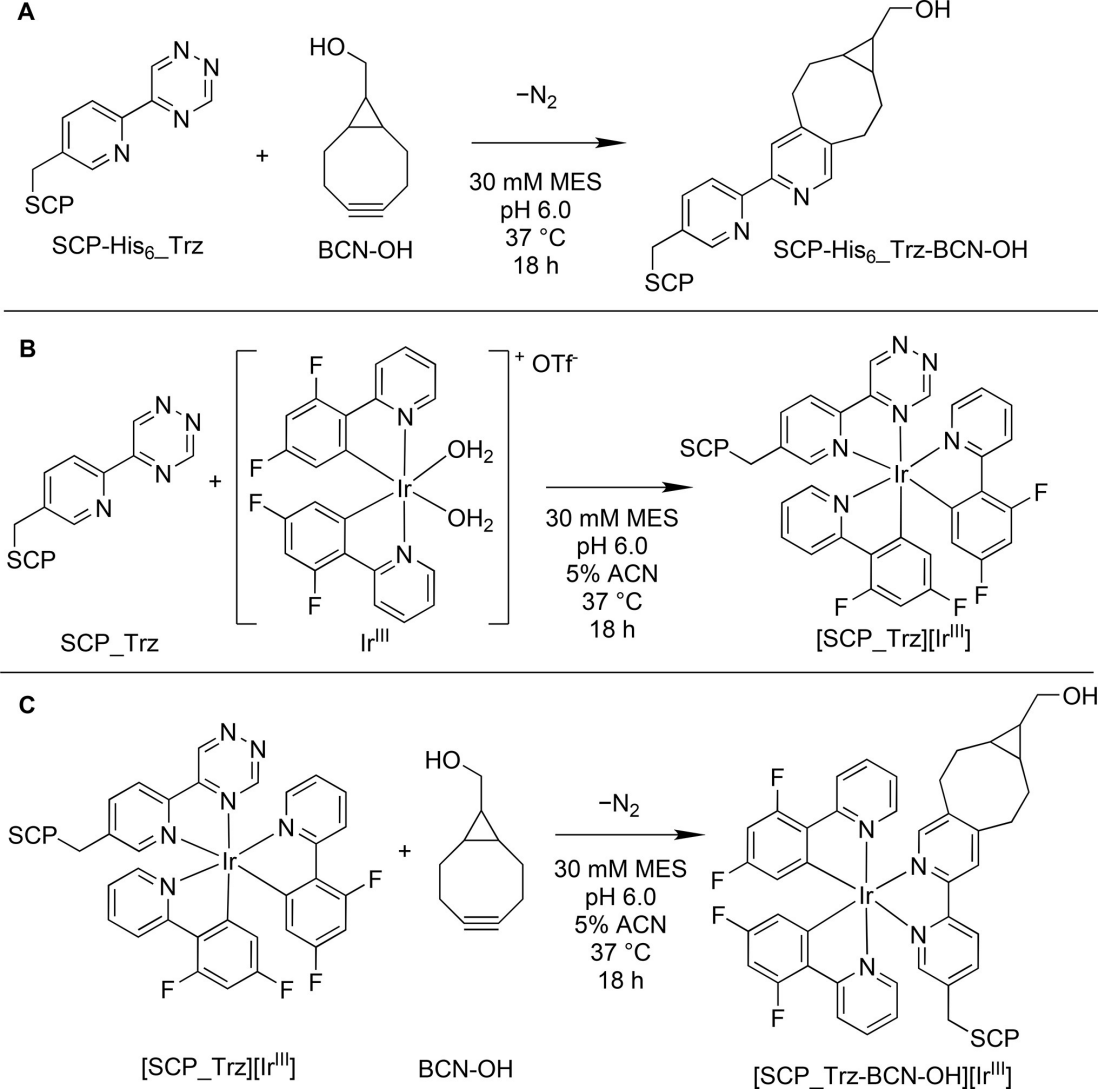
Bioorthogonal labelling reactions. A) Inverse‐electron‐demand Diels–Alder (IEDDA) reaction between SCP−His_6__Trz and BCN−OH to form SCP−His_6__Trz−BCN−OH. B) Iridium^III^ coordination by SCP_Trz to form [SCP_Trz][Ir^III^]. C) IEDDA reaction between [SCP_Trz][Ir^III^] and BCN−OH to form [SCP_Trz−BCN−OH][Ir^III^]. BCN−OH=bicyclo[6.1.0]non‐4‐yn‐9‐ylmethanol; Ir^III^=bis(aqua)bis(difluorophenylpyridine)iridium(III) triflate. Before Ir^III^ coordination, the N‐terminal methionine and C‐terminal His_6_‐tag (2093 Da total) were removed, giving SCP_Trz. Protein concentration was 10 μM in each reaction.

**Figure 3:**
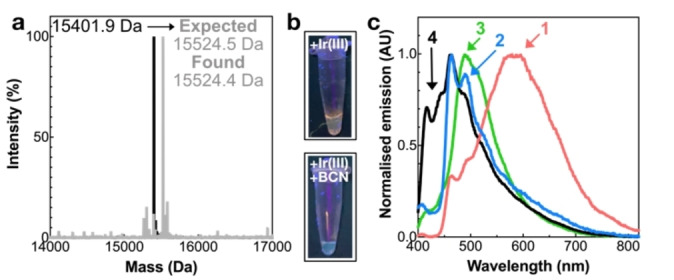
Bioorthogonal reactions. a) Deconvoluted mass spectrum of SCP−His_6__Trz before (black) and after (grey) labelling with BCN−OH. b) Optical images of [SCP_Trz][Ir^III^] (top) and [SCP_Trz−BCN−OH][Ir^III^] (bottom) under a UV lamp (*λ*=365 nm). c) Normalised emission spectra of [SCP_Trz][Ir^III^] (1, pink) and [SCP_Trz−BCN−OH][Ir^III^] (2, blue), [SCP_Bpy][Ir^III^] (3, green), and [wt‐SCP][Ir^III^] (4, black). Luminescence measurements were conducted in 30 mM MES, 5 % acetonitrile, pH 6.0, at 25 °C.

Previous studies using 5‐(pyridin‐2‐yl)‐1,2,4‐triazine have shown that it can coordinate a bis(difluorophenylpyridine) iridium(III) complex (simplified as Ir^III^ throughout the text), which could then catalyse the IEDDA reaction with BCN−OH to produce a luminescent product (see Figure S21 for data under aqueous conditions).^[27]^ Therefore, it was expected that the incorporated Trz would be able to bind Ir^III^ complexes (reaction B; Scheme 2) leading to luminescence upon BCN−OH addition offering the potential to produce switch‐on luminescent probes (reaction C; Scheme 2). To prevent non‐specific metal binding, the C‐terminal His_6_‐tag and N‐terminal methionine residue (Supporting Information Table 3) were cleaved to give SCP_Trz. After incubating 10 μM SCP_Trz for 18 h with 10× excess of Ir^III^ and removal of unbound metal, the resulting [SCP_Trz][Ir^III^] exhibited luminescence upon excitation with UV‐light, emitting an orange‐red colour (Figure 3b, top), in contrast to the previously reported small molecule complex, which was not emissive in organic solvents.^[27]^ Emission studies of [SCP_Trz][Ir^III^] excited at 360 nm revealed a large peak at *λ_max_* =590 nm (Figure 3c). As iridium complexes can bind to proteins through non‐specific hydrophobic interactions or through designed amino coordination,[^30^, ^40^] comparison between the [wild‐type(wt) SCP][Ir^III^] (*λ_max_* =465 nm) and [SCP_Trz][Ir^III^] indicated that the emission red‐shifted in the presence of Trz (Figure 3c), providing evidence that the desired Ir complex had formed. Unfortunately, we were unable to obtain LC–MS measurements of [SCP_Trz][Ir^III^] to verify stable Ir^III^ binding to Trz.

Finally, [SCP_Trz][Ir^III^] was incubated with BCN−OH to investigate whether an IEDDA reaction could proceed (reaction C; Scheme 2). A visible blue‐shift in emission was observed (Figure 3b, bottom), resulting from the transformation of the 1,2,4‐triazine ring into a pyridine during the IEDDA reaction,^[27]^ highlighting the ability of the iridium(III)‐bound Trz to act as a responsive luminescent probe. Although the excitation profile did not change compared to [SCP_Trz][Ir^III^] (Figure S22), emission studies of [SCP_Trz−BCN−OH][Ir^III^] revealed a hypsochromic shift to two peaks with *λ_max_* =510 and 460 nm, respectively (Figure 3c). The maximum at 460 nm corresponded to the emission profile of [wt‐SCP][Ir^III^] (Figure 3c), suggesting that some iridium was non‐specifically bound to SCP following the IEDDA reaction. Significantly, the 510 nm peak correlated with that of [SCP_BpyAla][Ir^III^] (Figure 3c and Figure S23), confirming production of the bipyridine ligand during the IEDDA in the presence of Ir^III^ and that the complex was still intact, analogous to the small molecule studies.^[27]^

In conclusion, a dual‐functional non‐canonical amino acid, Trz, was rationally designed for genetic code expansion, capable of participating in IEDDA reactions and coordinating metal complexes to a protein. Accordingly, a gene was designed featuring the TAG stop codon in a solvent‐exposed position of the sterol carrier protein (SCP) to incorporate Trz using amber stop‐codon suppression. Incorporation of Trz yielded a protein of the expected molecular weight that could undergo an IEDDA reaction with a bicyclononyne (BCN−OH), indicating the triazine moiety was accessible. Coordination to an iridium(III) complex revealed a luminescence mechanism upon Ir^III^ binding, which blue‐shifted upon reaction with BCN−OH. Whilst the relative rates of the IEDDA in the presence/absence of Ir^III^ were unable to be obtained, we expect the Ir(III) will play a similar role in catalysing the IEDDA reaction between SCP_Trz and BCN−OH as shown with the small molecule studies.^[27]^ Overall, Trz acts as a dual‐purpose amino acid combining metal binding properties and a bioorthogonal reaction handle as designed. We propose that the ability to double‐functionalise Trz to access modifiable luminescent probes provides a platform to develop tools to study protein–protein interactions or, more broadly, to advance multifunctional protein‐based therapeutics, where various practical moieties (fluorophores, drugs, radioisotopes, etc.) can be easily connected to a protein alongside a functional metal complex.

## Supporting Information

Electronic Supporting Information containing supplementary Figures and methods available online. Figure S1: ^1^H NMR of 4; Figure S2: ^13^C NMR of 4; Figure S3: DEPT 135 NMR of 4; Figure S4: ^1^H NMR of 5; Figure S5: ^13^C NMR of 5; Figure S6: DEPT 135 NMR of 5; Figure S7: ^1^H NMR of 6; Figure S8: ^13^C NMR of 6; Figure S9: DEPT 135 NMR of 6; Figure S10: ^1^H NMR of 7; Figure S11: ^13^C NMR of 7; Figure S12: ^1^H NMR of Trz; Figure S13: ^13^C NMR of Trz; Figure S14: ^1^H NMR of Trz‐Et; Figure S15: ^13^C NMR of Trz‐Et; Figure S16: DEPT 135 NMR of Trz‐Et; Figure S17: ^1^H NMR of BpyAla‐Et; Figure S18: ^13^C NMR of BpyAla‐Et; Figure S19: initial expression tests; Figure S20: SDS–PAGE of SCP−His_6_; Figure S21: luminescence of 1,2,4‐triazine small molecule complex controls; Figure S22: excitation spectra of [SCP_Trz][Ir^III^] and [SCP_Trz−BCN][Ir^III^]; and Figure S23: Luminescence and LC–MS of [SCP_BpyAla][Ir^III^].

## Conflict of Interests

The authors declare no conflict of interest.

## Supporting information

As a service to our authors and readers, this journal provides supporting information supplied by the authors. Such materials are peer reviewed and may be re‐organized for online delivery, but are not copy‐edited or typeset. Technical support issues arising from supporting information (other than missing files) should be addressed to the authors.

Supporting Information

## Data Availability

The data that support the findings of this study are available in the supplementary material of this article.
