# Supplementary material for: A Dual‐Purpose Non‐Canonical Amino Acid for the Expanded Genetic Code: Combining Metal‐Binding and Click Chemistry
Source: Angew Chem Int Ed Engl. 2024 Oct 31;63(52):e202413073. doi: 10.1002/anie.202413073 (PMC11656133; doi:10.1002/anie.202413073)
Supplement: Supplementary file 1 — Supporting Information [file ANIE-63-e202413073-s001.pdf]

Angewandte Chemie
Eine Zeitschrift der Gesellschaft Deutscher Chemiker

Supporting Information

A Dual-Purpose Non-Canonical Amino Acid for the Expanded Genetic Code: Combining Metal-Binding and Click Chemistry

G. J. Day, A. V. Zaytsev, R. C. Brewster, V. N. Kozhevnikov, A. G. Jarvis**

ESI

A Dual Purpose Non-Canonical Amino Acid for the Expanded Genetic Code: Combining Metal-Binding and Click Chemistry

Graham J. Day[a], Andrey V. Zaytsev[b]#, Richard C. Brewster[a]#, Valery N. Kozhevnikov[b]*, and Amanda G. Jarvis[a]*

[a] Dr G.J. Day, Dr R.C. Brewster, and Dr A.G. Jarvis
EaStCHEM School of Chemistry
University of Edinburgh
Joseph Black Building,
E-mail: amanda.jarvis@ed.ac.uk

[b] Dr A.V. Zaystev, and Dr V.N. Kozhevnikov
Department of Applied Sciences
Northumbria University
Newcastle-upon-Tyne, NE1 8ST
E-mail: valery.kozhevnikov@northumbria.ac.uk

# Contributed equally to this work.

ESI

Table of contents

Supplementary methods **3**

Noncanonical amino acid (ncAA) docking studies *3*

Protein expression tests *3*

Liquid chromatography–mass spectroscopy (LC–MS) *3*

Scale up protein expression and purification. *4*

Far-UV circular dichroism (CD) spectroscopy *5*

Protein modification *5*

Synthesis of noncanonical amino acids and ethyl ester derivatives *6*

Synthesis of 2 6

Synthesis of 3 7

Synthesis of 4 7

Synthesis of 5 8

Synthesis of Trz-Et 9

Synthesis of Trz 9

Synthesis of BpyAla-Et 10

Characterisation data **11**

Supplementary figures **29**

Protein sequences **31**

Supplememtary references **31**

Supplementary methods

Noncanonical amino acid (ncAA) docking studies

The ncAAs Trz and BpyAla were subjected to docking with the orthogonal tRNA/aminoacyl tRNA synthetase (aaRS) from *Methanococcus jannaschii* (PDB: 2PXH)[1] using FLARE™ (Cresset CADD software).

Protein expression tests

BL21 cells were transformed with pEVOL-BpyAla plasmid and p28 plasmid containing a gene for SCP (**Supplementary table 3**). A single colony was picked and grown in 10 mL LB media containing kanamycin (50 mg·L^{-1}) and chloramphenicol (35 mg·L^{-1}) at 37 ˚C overnight. The overnight culture was diluted 1 in 50 into fresh LB (10 mL) in 50 mL Falcon tubes. Cultures were grown to OD_{600} = 0.5 at 37 ˚C with shaking (200 rpm), at which point the ncAA was added (0.5 mM final concentration) and the temperature lowered to 30 ˚C. After 30 minutes, isopropyl β-D-1-thiogalactopyranoside (IPTG; 0.5 mM final concentration) and arabinose (0.02% final concentration) were added and the cultures incubated at 30 ˚C, 200rpm for 16 h. Cell pellets were harvested by centrifugation (4,122 × *g*, 15 minutes) and the supernatant discarded. Cells were re-suspended into phosphate buffered saline (PBS; 1 mL), transferred to a microcentrifuge tube and pelleted by centrifugation (18,000 × *g*, 5 minutes). The supernatant was discarded and cells re-suspended in primary amine free Bug Buster (0.5 mL; Merck, 70923) with DNAse (1 µL of a 20 mg·L^{-1} stock in H_2O) and spun on a tube rotator for 30 minutes at 30 rpm. Insoluble cell debris was removed by centrifugation (18,000 × g, 10 minutes) and the supernatant transferred to a clean microcentrifuge tube. Nickel-nitrilotriacetic acid (Ni-NTA) resin (~1 mL; Thermo Scientific) was conditioned into buffer A (30 mM Tris, 20 mM imidazole, 150 mM NaCl, pH 8) by centrifugation (1000 × *g*, 2 minutes), removal of supernatant, and re-suspension in fresh buffer 3 times. 100 µL of the resin solution was added to the cell lysate, which was incubated on a tube rotator for 30 minutes at 30 rpm. The resin was pelleted by centrifugation (1000 × *g*, 2 minutes) and supernatant discarded. The resin was washed 3 times by suspension in buffer A (1 mL) and pelleting by centrifugation (1000 × *g*, 2 minutes). After washing, buffer B (200 µL; 30 mM Tris, 330 mM imidazole, 150 mM NaCl, pH 8) was added and incubated on a tube rotator for 30 minutes at 30 rpm. The resin was pelleted by centrifugation (1000 × *g*, 5 minutes) and the supernatant withdrawn into a fresh tube. Analysis was performed by SDS–PAGE gel (NuPAGE 4-12% Bis-Tris gel) against a pre-stained molecular weight ladder (ABCAM ab116028).

Liquid chromatography–mass spectroscopy (LC–MS)

LC–MS (FT–ICR MS) experiments were performed using a Dionex Ultimate 3000 LC system (Thermo Scientific) connected in-line to a 12T SolariX XR FT-ICR mass spectrometer using the electrospray source (Bruker Daltonics). The purified protein solution (1 in 5 dilution, 5 µL) was injected onto a Proswift C4 RP-5H analytical column (500 µ x 10 cm; Thermo Scientific) and a

gradient of 2–70% B (mobile phases: A = water + 0.1% formic acid, B = acetonitrile + 0.1% formic acid), over 7 min, was employed. The data were acquired in broadband detection mode, using 2 megaword data points, over the m/z 147–3000 range. Bruker Data Analysis software was used to generate predicted spectra of the protein from the molecular formula.

Scale up protein expression and purification.

BL21 cells were transformed with pEVOL-BpyAla and p28 plasmid containing a gene for SCP-His_6 (**Supplementary table 3**). A single colony was picked and grown in 10 mL LB media containing kanamycin (50 mg·L^{-1}) and chloramphenicol (35 mg·L^{-1}) at 37 ˚C overnight. The overnight culture was diluted 1 in 50 into fresh Terrific broth (50 mL) in a baffled Erlenmeyer flask. Cultures were grown to OD_{600} = 0.5 at 37 ˚C with shaking (200 rpm), at which point the noncanonical amino acid Trz was added (0.5 mM final concentration) and the temperature lowered to 30 ˚C. After 30 minutes IPTG and arabinose were added (0.5 mM and 0.02% final concentrations, respectively) and the cultures left at 30 ˚C/200rpm for 16 h. Cell pellets were harvested by centrifugation (4,122 × *g*, 15 minutes) and the supernatant discarded. Cells were re-suspended into PBS (10 mL), transferred to a 15 mL tube and pelleted by centrifugation (4,122 × *g*, 15 minutes). The supernatant was discarded, and the cell pellet stored at −20 °C until use.

To purify, the pellet was thawed on ice, then re-suspended in lysis buffer (10 mL; 50 mM Tris·HCl, 50 mM imidazole, 150 mM NaCl, pH 8) and DNAse (1 mg) were added. Cells were disrupted by sonication (2 s pulses, 90% power) for 3 minutes. The cell extract obtained after centrifugation (1 h, 18000 × *g*) was filtered with a 0.4 µm syringe filter and loaded onto a Ni–NTA column (1 mL, HisTrap FF) equilibrated with wash buffer (30 mM Tris·HCl, 50 mM imidazole, 150 mM NaCl, pH 8) at a flow of 1 mL·min^{-1}. The column was washed with 10 column volumes of wash buffer before being eluted with elution buffer (30 mM Tris·HCl, 330 mM imidazole, 150 mM NaCl, pH 8). The protein solution was dialysed against 1 L of buffer (30 mM MES, pH 6) at 4 °C, then concentrated using an Amicon Ultra 15 10 kDa centrifugal filter unit. Protein concentrations were estimated *via* UV-vis spectrophotometry (Denovix DS-11). Analysis was performed by SDS–PAGE gel (NuPAGE 4-12% Bis-Tris gel) against a pre-stained molecular weight ladder (BioRad Plus Protein Kaleidoscope).

Wild-type SCP-His_6 was expressed as above using BL21 *E. coli* transformed with the p28 plasmid carrying the *scp* gene. Cells were grown to OD_{600} 0.5 of in LB media containing kanamycin (50 mg·L^{-1}) and protein expression was induced by the addition of 0.5 mM IPTG. Cells were harvested and purified as described above.

The yield of protein was calculated by determining the mass of purified protein by measuring the final concentration via UV-vis spectroscopy (Nanodrop) using the Beer–Lambert law (Equation 1), where *c* is protein concentration (M), A_{280} is the absorbance at 280 nm, and ε_{280} is the extinction coefficient at 280 nm of SCP_BpyAla (28680 M^{-1}·cm^{-1})[3]. Based on the similarity of the extinction coefficients of bypridine and 5-pyridyl-1,2,4-triazine in MeCN (12468 ± 527 M^{-1}cm^{-1} vs 12045 ± 373

$M^{-1}cm^{-1}$), the extinction coefficient of the SCP_Trz protein was assumed to be the same as the SCP_BpyAla. Subsequently, this value was multiplied by the protein molecular weight (14604 kDa) to convert the concentration to milligrams of protein per millilitre of solution. After multiplying by the volume of the solution, the total number of milligrams of purified protein per 50 mL expression culture was known, which could be converted to $mg \cdot L^{-1}$ by multiplying by 20.

$$c = \left(\frac{A_{280}}{\varepsilon_{280}}\right) \quad \text{Equation 1}$$

SCP-His$_6$_Trz and wild-type SCP-His$_6$ had their *N*- and *C*-termini cleaved using Tobacco Etch Virus (TEV) protease following purification and before dialysis into 30 mM MES buffer. Instead, the His$_6$-tagged proteins were prepared for TEV-mediated cleaved by dialysing against 1 L lysis buffer for 18 h, 4°C. Proteins were then cleaved by incubation for 18 h with 0.014 equivalents of TEV protease with final concentratiosn of 1 mM dithiothreitol and 0.5 mM ethylenediaminetetraacetic acid. Subsequently, the mixture was loaded onto a Ni–NTA column (1 mL, HisTrap FF) equilibrated with wash buffer 2 (30 mM Tris·HCl, 50 mM imidazole, 150 mM NaCl, pH 8) at a flow of 1 $mL \cdot min^{-1}$. The flow-through containing the cleaved protein was collected and dialysed against 1 L of buffer (30 mM MES, pH 6) at 4 °C, then concentrated using an Amicon Ultra 15 10 kDa centrifugal filter unit, as above.

Far-UV circular dichroism (CD) spectroscopy

Protein samples were measured in buffer (30 mM MES, pH 6.0) and loaded into a CD-quartz cuvette (pathlength 0.5 mm) under the provided settings (**Supplementary table 1**). Data were normalised to maximum intensity (millidegrees) to compare 222/208 nm ratios.

Supplementary table 1. Settings for far-UV circular dichroism spectroscopy.

Pathlength (mm)	0.5
Time-per-point (s)	5
Number of scans averaged	3
Range (nm)	200–260
Temperature (°C)	20

Protein modification

Bicyclo[6.1.0]non-4-yn-9-ylmethanol (BCN-OH) was prepared in dimethylsulfoxide (DMSO) to a concentration of 1 mM. An Ir^{III} ($[Ir(dfppy)_2(OH_2)_2]^+CF_3SO_3^-$) solution was prepared in acetonitrile to a concentration of 1 mM. Protein solution (20 µL, 10 µM) was added to a PCR tube containing 1.5 µL of BCN-OH (approx. 10 eq.). The solutions were incubated for 18 h at 37 °C. To remove excess Ir^{III}, the samples containing Ir^{III}, excess N-methylimidazole (NMI; 1 µL; 50% v/v in H_2O) was added and incubated for 1 h at 37 °C, then desalted (Zeba™ Spin Desalting Column, 7K MWCO;

ThermoFisher Scientific), following the manufacturer's protocol. The sample was then split and BCN-OH (1 µL; 1 mM in DMSO) was added to one of the samples and incubated for 4 h at 37 °C. BCN-OH-labelled proteins were analysed *via* LC–MS, as above. Ir^{III}-labelled proteins were analysed *via* fluorescence spectroscopy (CLARIOstar Plus; BMG LABTECH). Samples were aliquoted into black-walled 96-well plates and measured under the provided settings (**Supplementary table 2**).

Supplementary table 2: Settings for fluorescence experiments.

**Excitation**		**Emission**	
Number of flashes per well	100	*Number of flashes per well*	100
Excitation bandwidth (nm)	10	*Excitation bandwidth (nm)*	16
Emission wavelength (nm)	520	*Excitation wavelength (nm)*	360
Emission bandwidth (nm)	16	*Emission bandwidth (nm)*	10
Optic used	Top	*Optic used*	Top
Temperature (°C)	20	*Temperature (°C)*	20

Synthesis of noncanonical amino acids and ethyl ester derivatives

All solvents and reagents were purchased from commercial suppliers and used without further purification unless otherwise noted. NMR spectra were recorded on a JEOL ECS400FT Delta spectrometer (399.78 MHz for 1H NMR, 100.53 MHz for 13C NMR) or a Bruker Avance III 500 MHz spectrometer (500 MHz for 1H NMR , 126 MHz for 13C NMR). Chemical shifts are reported in parts per million (ppm) relative to a tetramethylsilane internal standard. High resolution mass spectra were recorded using electrospray on a Bruker MicroTOF II mass spectrometer. Bipyridylalanine was prepared according to literature procedures.[2]

Synthesis of Trz closely followed a previously reported protocol for 5-(pyridine-2-yl)-1,2,4-triazine.[3] Described in detail below.

Synthesis of 2

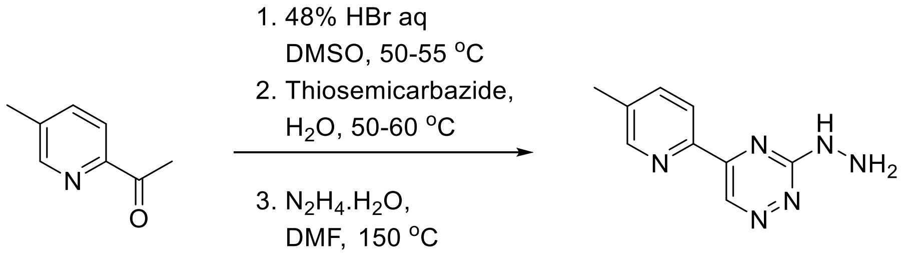

A 47% HBr aqueous solution (12.5 mL) was added to a solution of 2-acetyl-5-methylpyridine (4.70 g, 34.8 mmol) in DMSO (37 mL) portion-wise to keep the temperature of the reaction bellow 50 ˚C. After completion of the addition the reaction mixture was stirred at 50-55 ˚C for 20 h. Water (50 mL) was added and the mixture was neutralised by addition of Na_2CO_3 (careful CO_2 evolution!). Thiosemicarbazide (3.16 g, 34.8 mmol) and more Na_2CO_3 (3.62 g, 34.8 mmol) were added and the mixture was stirred at RT for 10 minutes. The mixture was then warmed up to 50–60 °C and stirred at this temperature for 1 hour. The mixture was then filtered and the filtrate was neutralised by careful addition of acetic acid. The resulting orange solid was filtered off, washed with water and dried in avacuum oven at 40 ˚C for 3 hours to give 7.09 g. This product was used without further purification. To a suspension of the product from the previous step (3.58 g) in DMF (10 mL) heated to 120 ˚C, $N_2H_4 \cdot H_2O$ (2.56 mL) was added upon stirring and the reaction mixture was heated to 150 ˚C for 30 min. The content of the flask was cooled to room temperature and the resulted precipitate was filtered off and washed with ethanol (1 mL), water (10 mL) and pet.ether. The solid was dried in a vacuum oven at 50 ^{0}C to give the desired product (1.56 g, 44%) as a yellow solid.

^{1}H NMR (methanol-*d4*, 400 MHz) 9.38 (s, 1H, triazine-H), 8.50 (app. d, 1H, pyr-H), 8.33 (app. d, 1H, pyr-H), 7.70 (app. t, 1H, pyr-H), 2.40 (s, 3H, CH_3)

^{13}C NMR (methanol-*d4*, 101 MHz) 164.6 (quat.), 156.4 (quat.), 151.0 (CH), 150.2 (quat.), 138.9 (CH), 138.7 (CH), 138.2 (quat.), 123.3 (CH), 18.7 (CH_3)

Synthesis of 3

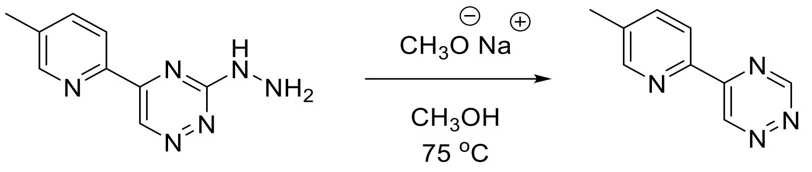

Compound **2** (799 mg, 3.95 mmol) was added to a solution of MeONa in methanol (prepared from Na (909 mg, 39.9 mmol) and methanol (80 mL)) and the reaction mixture was refluxed under dry atmosphere for 18 h. The flask was cooled to room temperature, acidified with acetic acid (4 mL) and water (80 mL) was added. The organics were extracted with DCM (3 × 30 mL); the combined organic layers were dried over $MgSO_4$ and volatiles were removed on a rotary evaporator. The residue was subjected to a flash column chromatography using pet.ether: AcOEt = 100:0 → 50:50 as an eluent to give **3** (445 mg, 65%) as a yellow solid.

^{1}H NMR ($CDCl_3$, 400 MHz) 10.25 (d, 1H, *J* = 1.9 Hz, triazine-H), 9.67 (d, 1H, *J* = 1.9 Hz, triazine-H), 8.61 (s, 1H, pyr-H) 8.42 (d, 1H, *J* = 8.2 Hz, pyr-H), 7.70 (d, 1H, *J* = 8.2 Hz, pyr-H), 2.46 (s, 3H, CH_3)

ESI
^{13}C NMR ($CDCl_3$, 101 MHz) 157.3 (CH), 154.1 (quat.), 150.8 (CH), 149.1 (quat.), 147.4 (CH), 138.0 (CH), 137.3 (quat.), 122.6 (CH), 18.9 (CH_3).
HRMS (m/z): (ESI) calc. for $C_9H_9N_4$ $[M+H]^+$: 173.08217; found: 173.08210.

Synthesis of 4

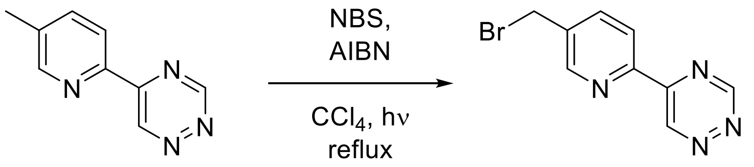

A solution containing **3** (344 mg, 2.00 mmol), N-bromosuccinimide (249 mg, 1.40 mmol) and AIBN (25 mg, 0.152 mmol) in CCl_4 (30 mL) was heated to reflux under irradiation with a 30 W halogen bulb for 1 h. The solvent was removed under reduced pressure and the residue was suspended in DCM (30 mL), filtered off, washed with DCM and discarded. DCM was removed on a rotary evaporator and the residue was subjected to a flash column chromatography using DCM:Et_2O = 100:0 → 85:15 as an eluent to give **4** (154 mg, 44%) as a yellow solid.
^1H NMR ($CDCl_3$, 400 MHz) 10.26 (d, 1H, J = 2.4 Hz, triazine-H), 9.71 (d, 1H, J = 2.4 Hz, triazine-H), 8.77 (s, 1H, pyr-H) 8.52 (d, 1H, J = 7.9 Hz, pyr-H), 7.93 (dd, 1H, J = 8.2 and 2.0 Hz, pyr-H), 4.53 (s, 2H, CH_2)
^{13}C NMR ($CDCl_3$, 101 MHz) 157.3 (CH), 153.4 (quat.), 151.5 (quat.), 150.2 (CH), 147.4 (CH), 138.1 (CH), 137.0 (quat.), 122.9 (CH), 28.8 (CH_2)
HRMS (m/z): (ESI) calc. for $C_9H_8N_4{}^{79}Br$ $[M+H]^+$: 250.99430; found: 250.99268.

Synthesis of 5

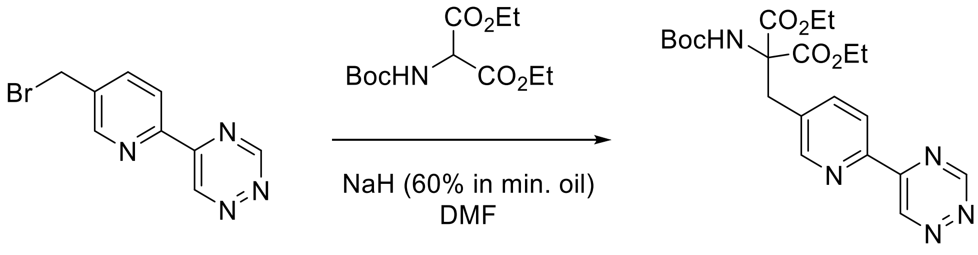

Diethyl (Boc-amino)malonate (125 mg, 116 µL, 454 µmol) was dissolved in anhydrous DMF (6 mL) and NaH (60% in mineral oil; 18 mg, 454 µmol) was added and the mixture was stirred at room temperature for 1 h. Compound **4** (95 mg, 378 µmol) was added in one portion and the reaction

mixture was stirred at room temperature under Ar atmosphere for 20 h. Water (50 mL) was added and the organics were extracted with diethyl ether (3 × 20 mL). The combined organic layers were washed with water (2 × 15 mL), saturated brine (15 mL) and dried over $MgSO_4$. The volatiles were removed under reduced pressure, and the residue was purified by a flash column chromatography using DCM:AcOEt = 100:0 → 50:50 as an eluent to give **5** (129 mg, 77%) as a yellow oil.

^{1}H NMR ($CDCl_3$, 400 MHz) 10.26 (d, 1H, *J* = 2.1 Hz, triazine-H), 9.69 (d, 1H, *J* = 2.1 Hz, triazine-H), 8.48 (d, 1H, *J* = 2.2 Hz, pyr-H), 8.45 (d, 1H, *J* = 8.0 Hz, pyr-H), 7.61 (dd, 1H, *J* = 8.0 and 2.2 Hz, pyr-H), 5.81 (s, 1H, N*H*Boc), 4.35 – 4.20 (m, 4H, OCH_2CH_3), 3.75 (s, 2H, CH_2), 1.49 (s, 9H, $C(CH_3)_3$), 1.30 (t, 6H, *J* = 7.1 Hz, OCH_2CH_3)

^{13}C NMR ($CDCl_3$, 101 MHz) 167.3, 157.3, 154.2, 153.9, 151.4, 150.6, 147.4, 139.1, 135.0, 122.5, 81.0, 66.9, 63.1, 35.9, 28.4, 14.2.

HRMS (m/z): (ESI) calc. for $C_{21}H_{28}O_6N_5$ $[M+H]^+$: 446.20340; found: 446.20341.

Synthesis of Trz-Et

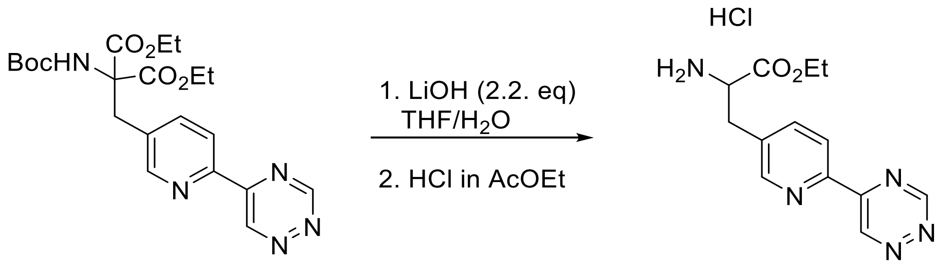

A solution of 1 M LiOH (556 µL, 556 µmol) was added to **5** (109 mg, 245 µmol) dissolved in THF (3 mL) and the reaction mixture was stirred a room temperature for 20 h. 2 M HCl (279 µL, 556 µmol) was added and the volatiles were removed under reduced pressure. The dry solid was sonicated with $HCl_{sat.}$ in AcOEt (2 mL) and concentrated. The residue was subjected to reverse phase column chromatography using water (containing 1% AcOH):acetonitrile (containing 1% AcOH) = 100:0 → 80:20 as an eluent to give Trz-Et (44 mg, 58%) as a beige solid.

^{1}H NMR (DMSO-*d*$_6$, 400 MHz) 10.19 (d, 1H, *J* = 2.1 Hz, triazine-H), 9.85 (d, 1H, *J* = 2.1 Hz, triazine-H), 8.69 (d, 1H, *J* = 2.2 Hz, pyr-H), 8.43 (d, 1H, *J* = 8.1 Hz, pyr-H), 7.95 (dd, 1H, *J* = 8.1 and 2.2 Hz, pyr-H), 4.07 (q, 2H, *J* = 7.1 Hz, OCH_2CH_3), 3.67 (dd, 1H, *J* = 7.8 and 5.9 Hz, CH), 3.03 (dd, 1H, *J* = 13.6 and 5.9 Hz, CH_aH_b), 2.92 (dd, 1H, *J* = 13.6 and 7.8 Hz, CH_aH_b), 1.14 (t, 3H, *J* = 7.1 Hz, OCH_2CH_3).

^{13}C NMR (DMSO-*d*$_6$, 101 MHz) 174.5 (quat.), 157.3 (CH), 153.4 (quat.), 151.1 (CH), 149.2 (quat.), 146.9 (CH), 138.7 (CH), 137.8 (quat.), 122.1 (CH), 60.3 (CH_2), 55.1 (CH), 37.3 (CH_2), 14.1 (CH_3).

HRMS (m/z): (ESI) calc. for $C_{13}H_{16}O_2N_5$ $[M-Cl]^+$: 274.12900; found: 274.12985.

Synthesis of Trz

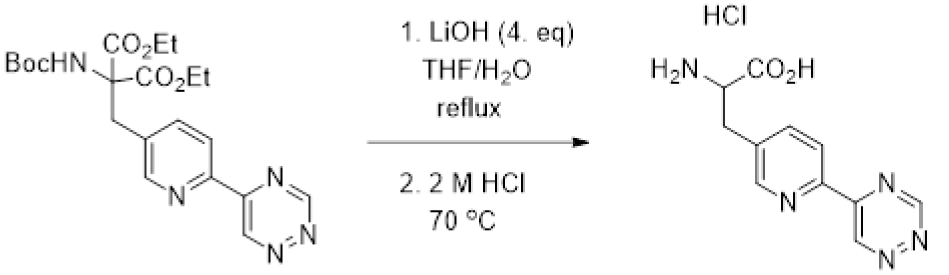

A solution of 1 M LiOH (1.03 mL, 1.03 mmol) was added to **5** (115 mg, 0.26 mmol) dissolved in MeOH (4 mL) and the reaction mixture was heated to reflux for 20 h under argon. Methanol was removed under reduced pressure, and the residue was heated at 70 °C for 2 h with 2 M HCl (3 mL). The volatiles were removed under reduced pressure to give Trz (NMR showed full conversion, the sample contains LiCl 1.03 mmol).

^{1}H NMR (DMSO-d_6, 400 MHz) 10.21 (s, 1H, triazine-H), 9.89 (d, 1H, triazine-H), 8.79 (d, 1H, pyr-H), 8.76 (br s, 3H, $^{+}NH_3$), 8.47 (d, 1H, J = 8.0 Hz, pyr-H), 8.06 (d, 1H, J = 8.0 Hz, pyr-H), 4.32 – 4.27 (m, 1H, CH), 3.36 (d, 1H, J = 6.2 Hz, CH_2).

^{13}C NMR (DMSO-d_6, 101 MHz) 170.0 (quat.), 157.4 (CH), 153.3 (quat.), 151.4 (CH), 149.9 (quat.), 147.0 (CH), 139.4 (CH), 135.2 (quat.), 122.5 (CH), 52.7 (CH), 32.7(CH_2).

HRMS (m/z): (ESI) calc. for $C_{11}H_{12}O_2N_5$ $[M\text{-}Cl]^+$: 246.09855; found: 246.0979.

Synthesis of BpyAla-Et

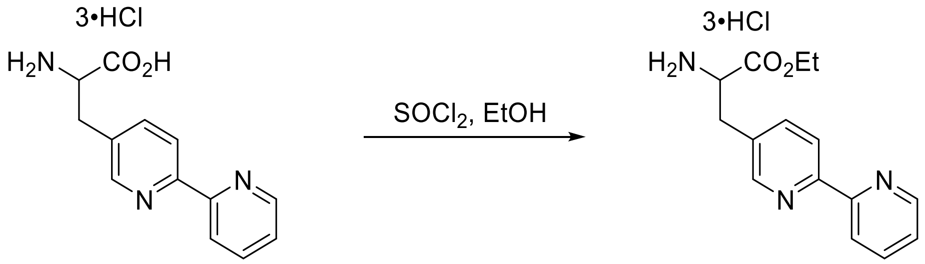

2,2-Bipyridylalanine (350 mg, 1.0 mmol) was suspended in EtOH (20 mL) and cooled on ice. $SOCl_2$ (0.15 mL, 2.0 mmol) was added dropwise to the solution, which was then heated at reflux for 3 h. After cooling solution was concentrated *in vacuo* giving BpyAla-Et as a beige solid (380 mg, quantitative yield).

^{1}H NMR (500 MHz, D_2O): 8.89 (d, 1H, J = 5.7 Hz), 8.82 (s, 1H), 8.68 (t, 1H, J = 7.9 Hz), 8.63 (d, 1H, J = 8.2 Hz), 8.39 (d, 1H, J = 9.3 Hz), 8.19 (d, 1H, J = 8.1 Hz), 8.09 (t, 1H, J = 6.0 Hz), 4.59 (t, 1H, J = 7.2 Hz), 4.36 – 4.24 (m, 2H), 3.53 (d, 2H, J = 7.1 Hz), 1.23 (d, 3H, J = 8.4 Hz);

ESI

^{13}C NMR (126 MHz, D_2O) δ 168.9 (C), 149.9 (CH), 146.4 (CH), 141.0 (CH), 134.2 (C), 127.1 (CH), 124.5 (CH), 123.2 (CH), 63.8 (CH_2), 53.2 (CH), 32.7 (CH_2), 13.1 (CH_3);

HRMS (m/z): (ESI) calc. for $C_{15}H_{16}O_2N_3$ $[M-H]^+$: 272.13935; found: 272.14050.

Characterisation data

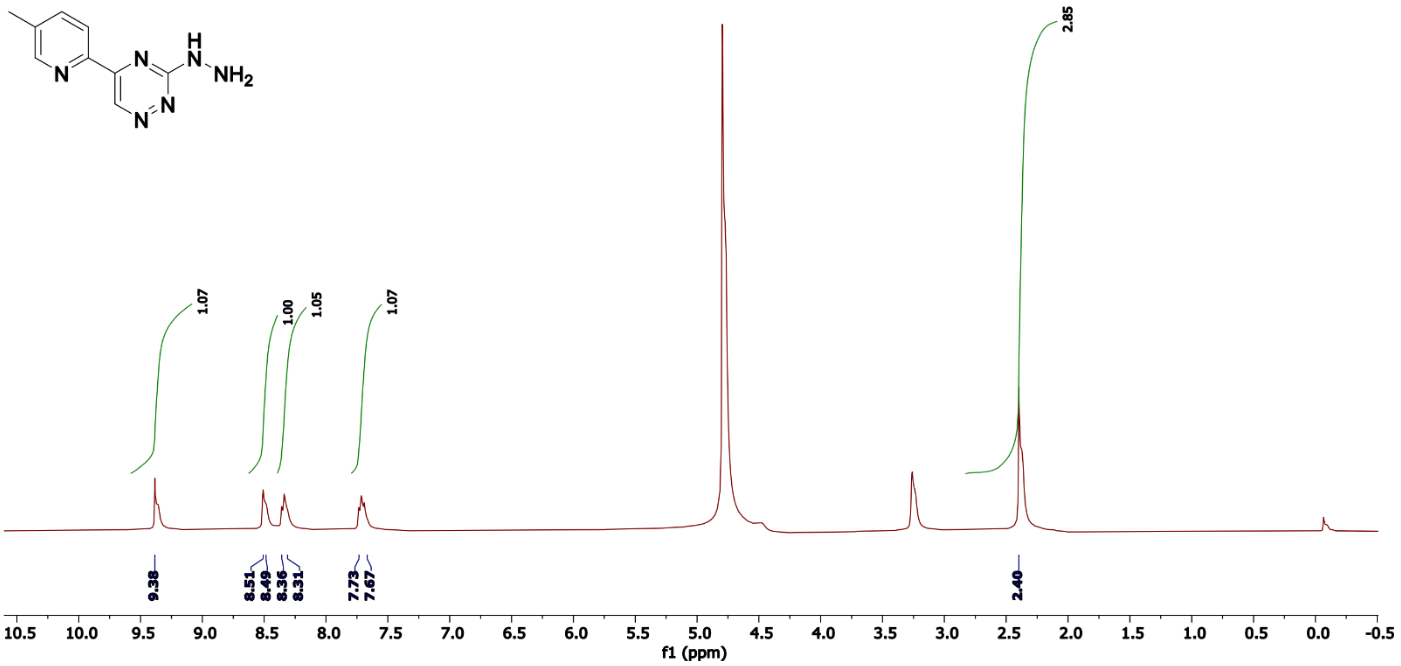

Figure S1. ^{1}H NMR spectrum of **4** in CD_3OD

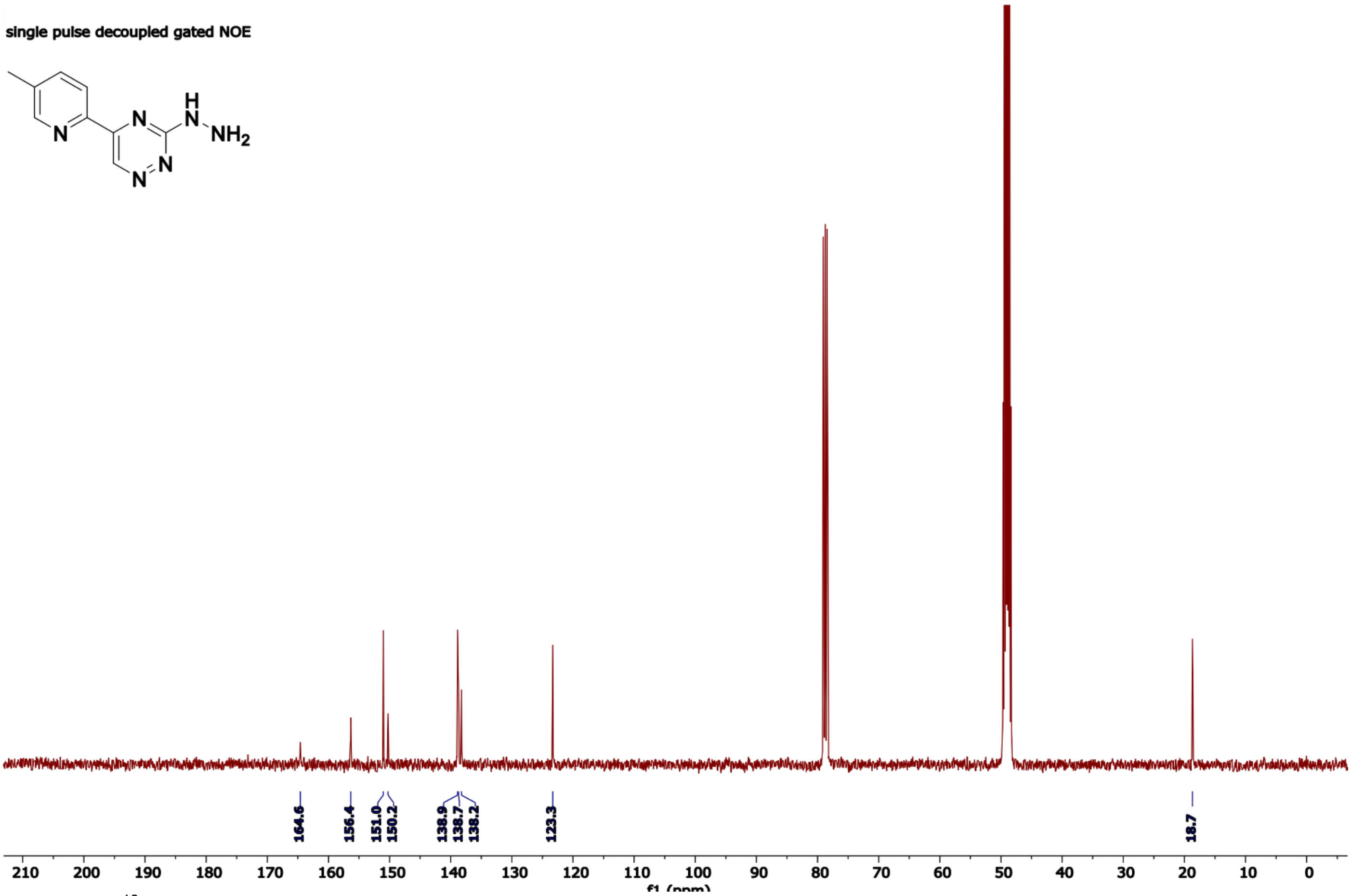

Figure S2. ^{13}C NMR spectrum of **4** in CD_3OD.

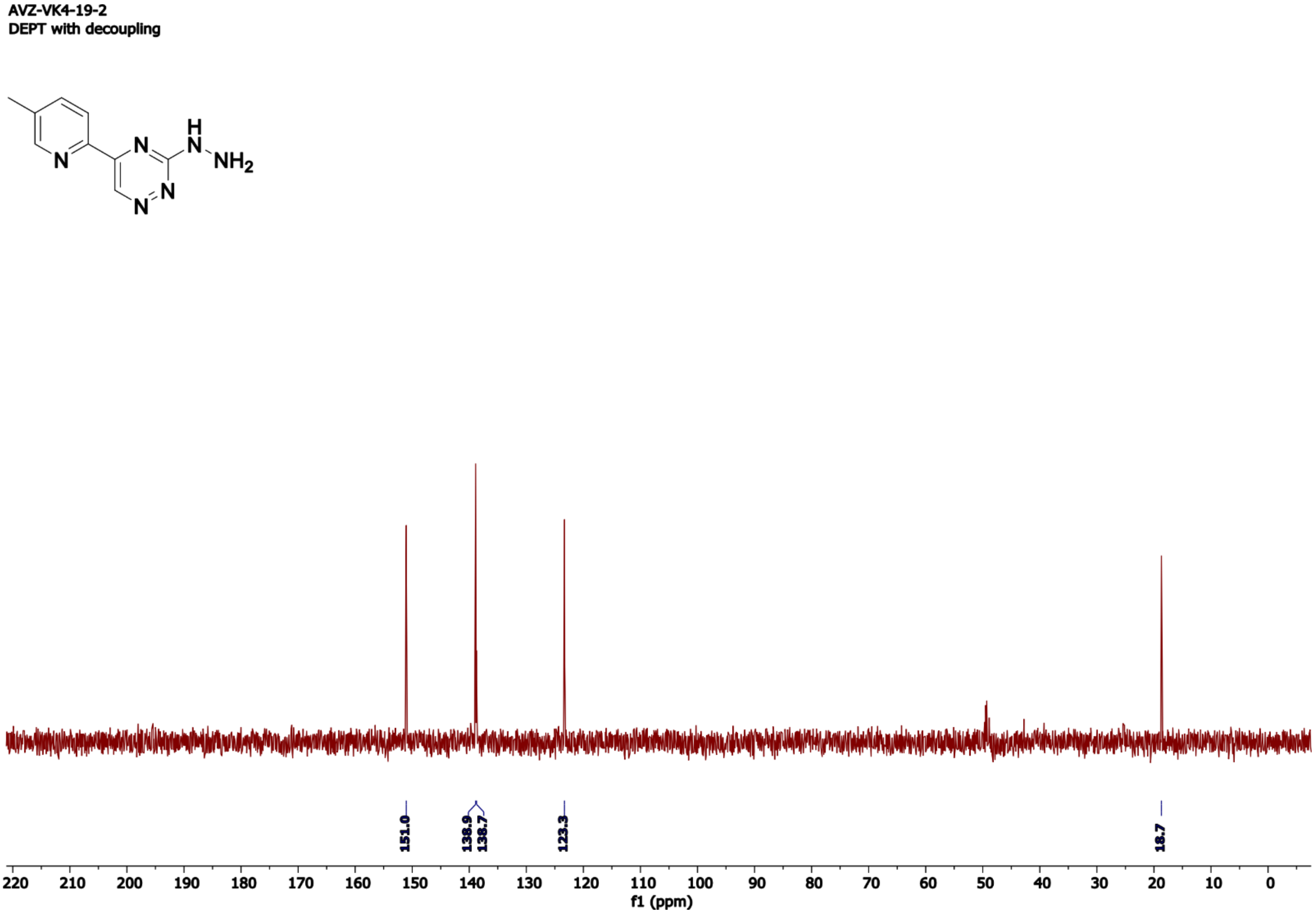

Figure S3. DEPT135 NMR spectrum of **4** in CD_3OD.

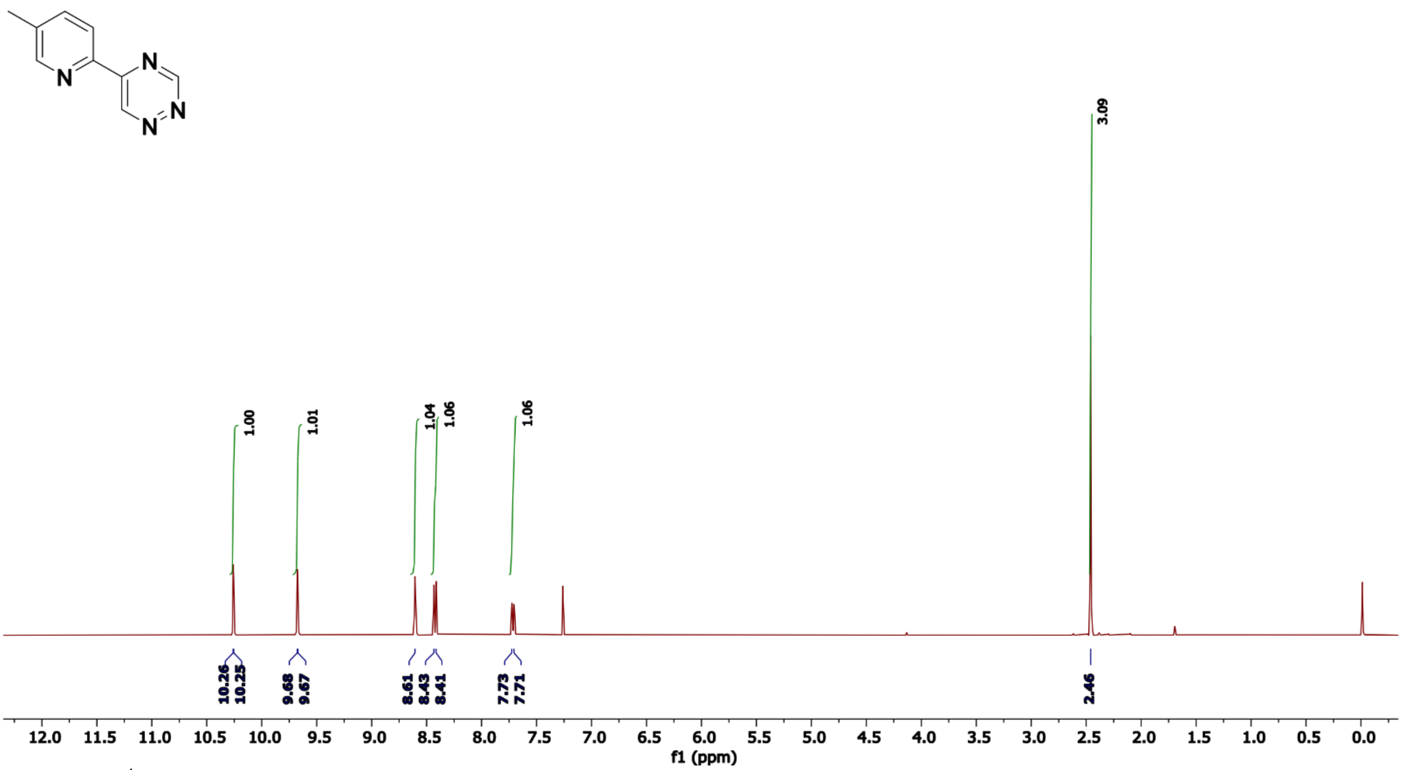

Figure S4. ^{1}H NMR spectrum of **5** in $CDCl_3$.

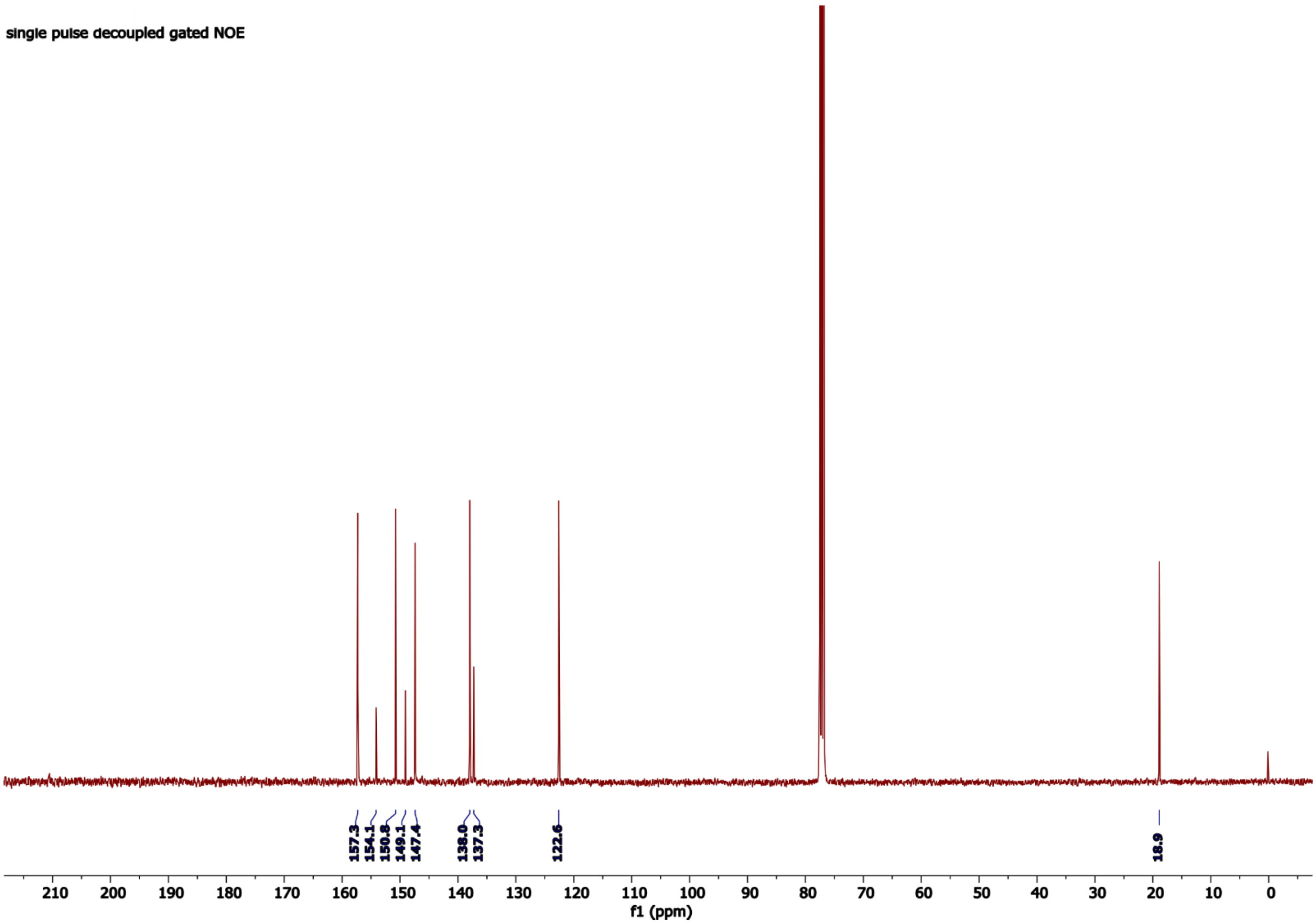

Figure S5. ^{13}C NMR spectrum of **5** in $CDCl_3$.

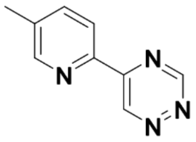

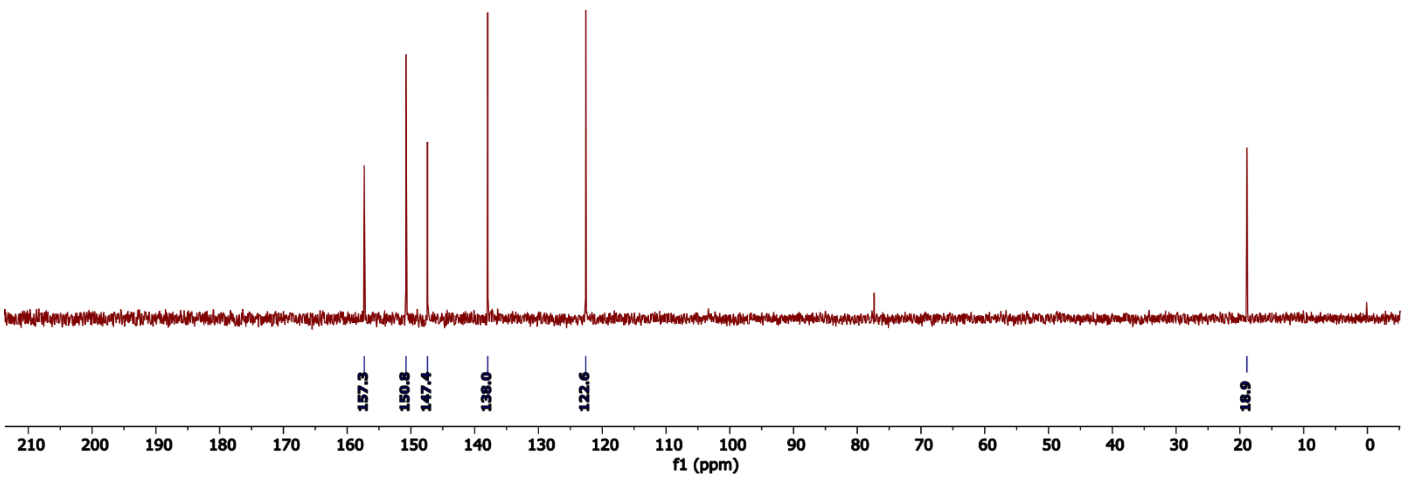

Figure S6. DEPT135 NMR spectrum of **5** in $CDCl_3$.

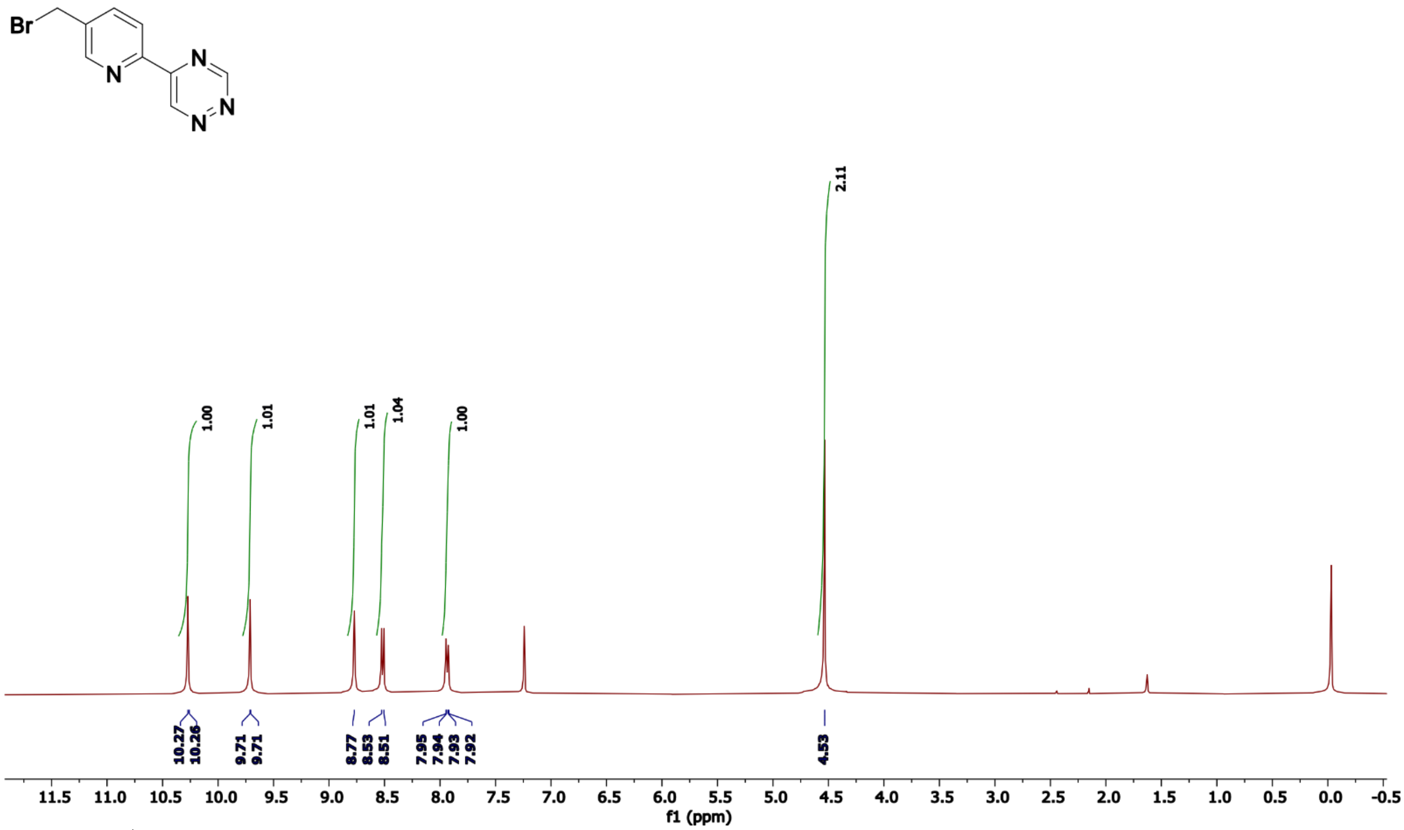

Figure S7. ^{1}H NMR spectrum of **6** in $CDCl_3$.

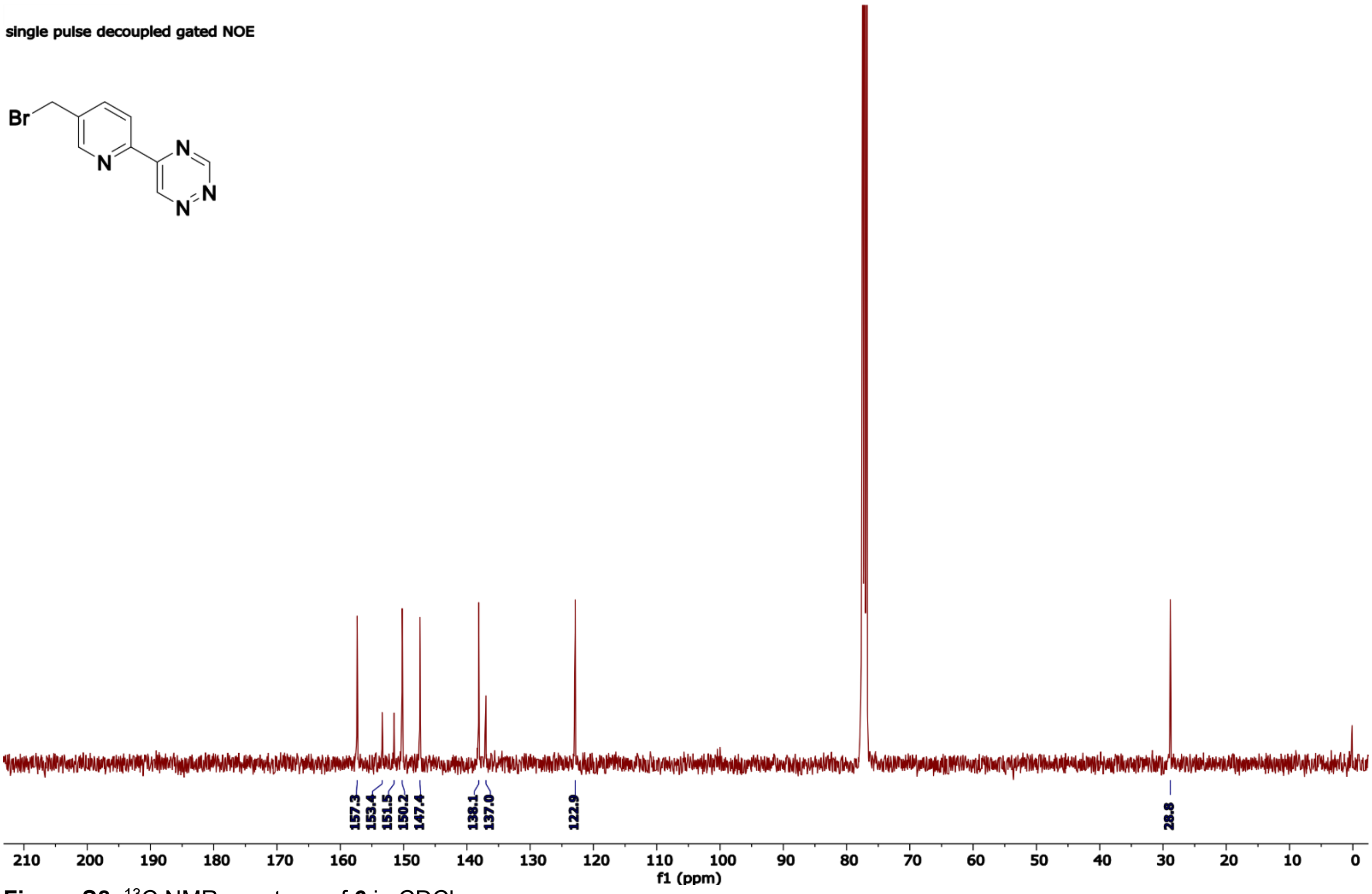

Figure S8. ^{13}C NMR spectrum of **6** in $CDCl_3$.

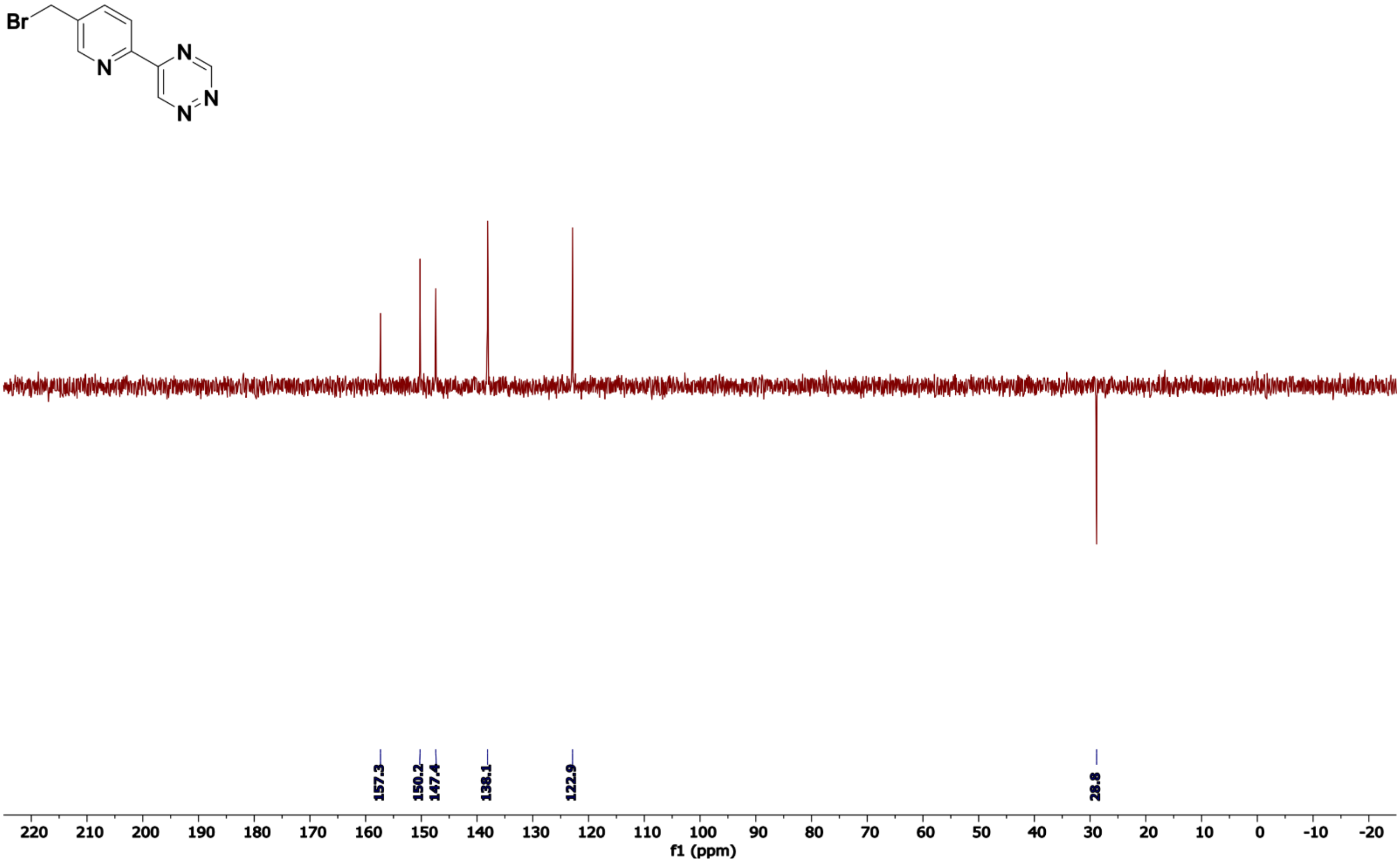

Figure S9. DEPT135 NMR spectrum of **6** in $CDCl_3$.

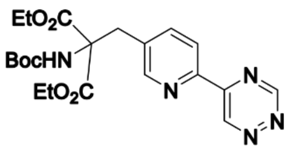

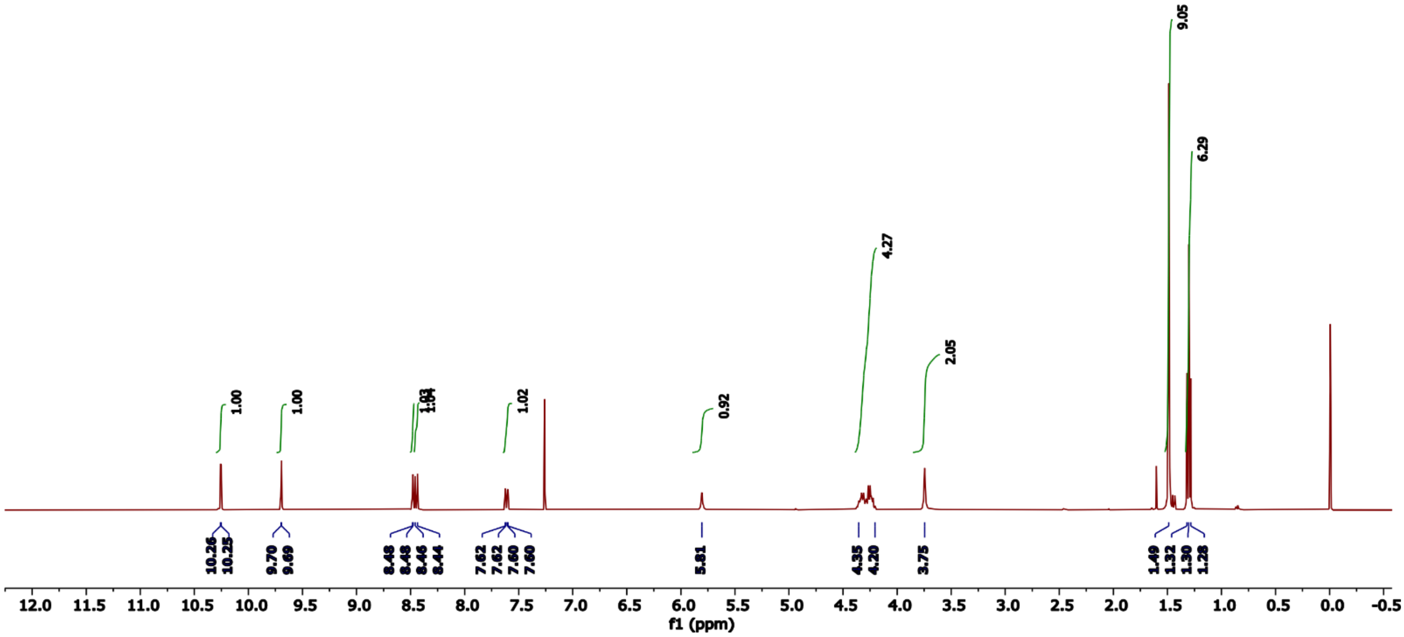

Figure S10. ^{1}H NMR spectrum of **7** in $CDCl_3$.

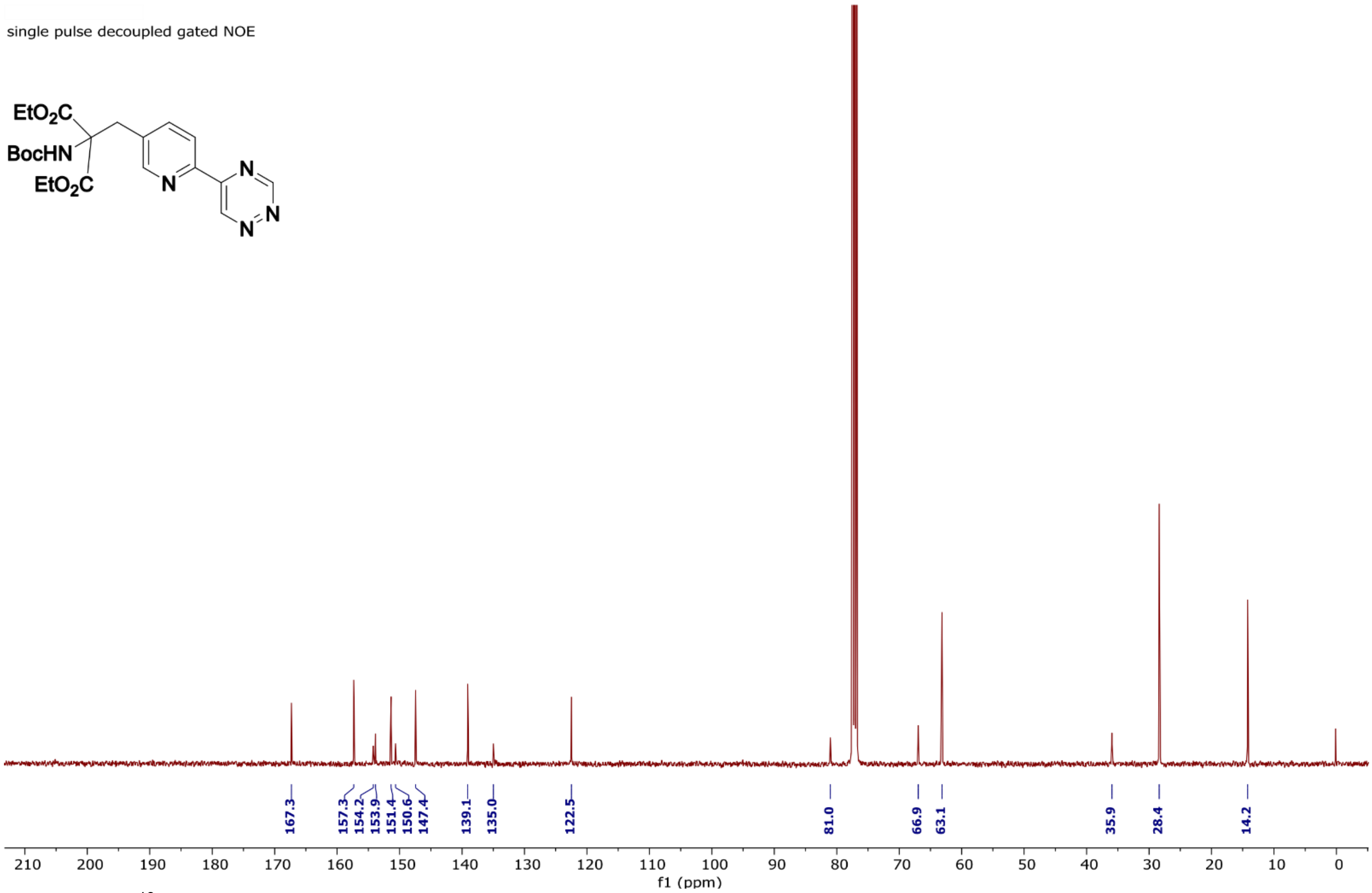

Figure S11. ^{13}C NMR spectrum of **7** in $CDCl_3$.

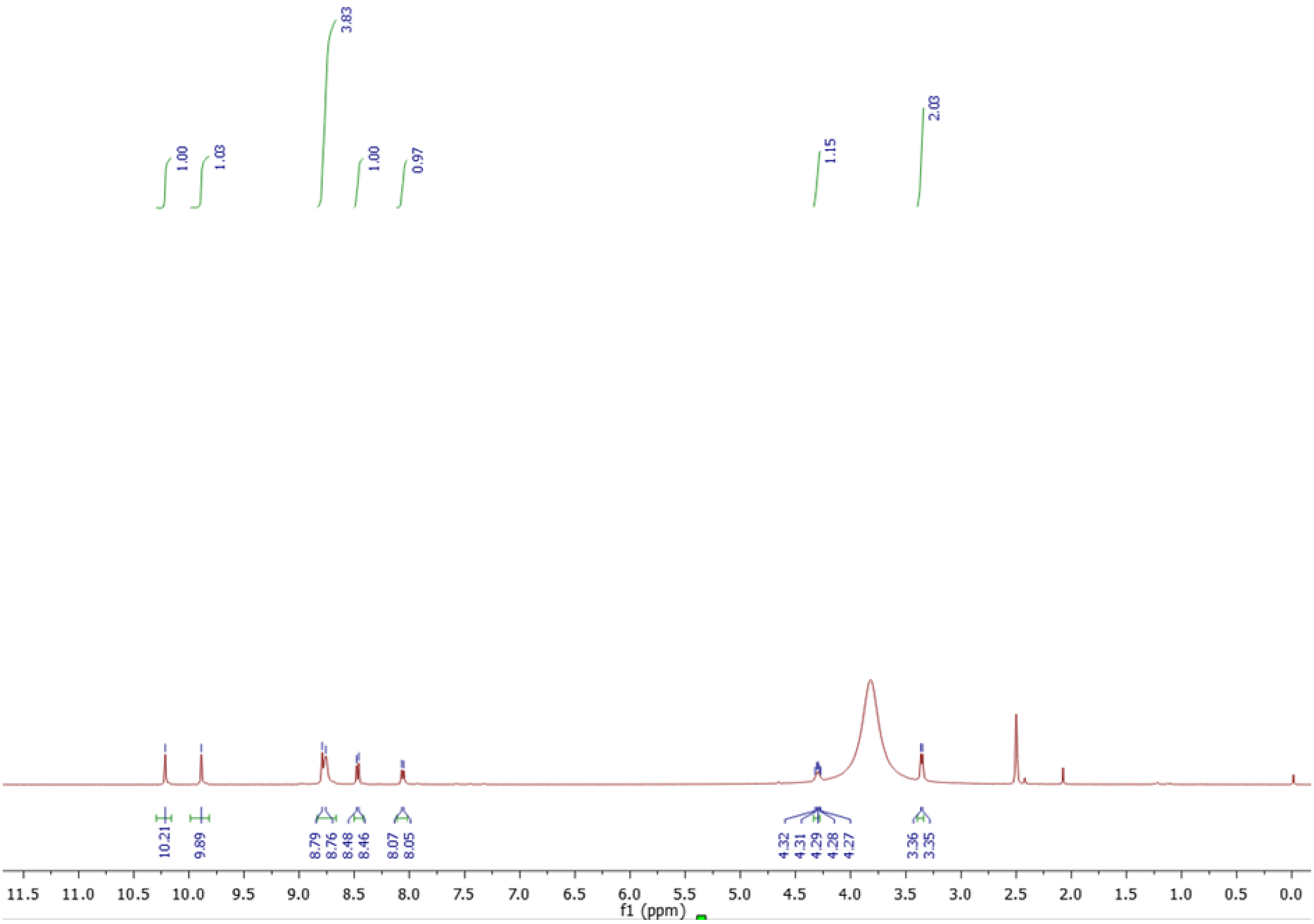

Figure S12. ^{1}H NMR spectrum of **Trz** in DMSO-d_6.

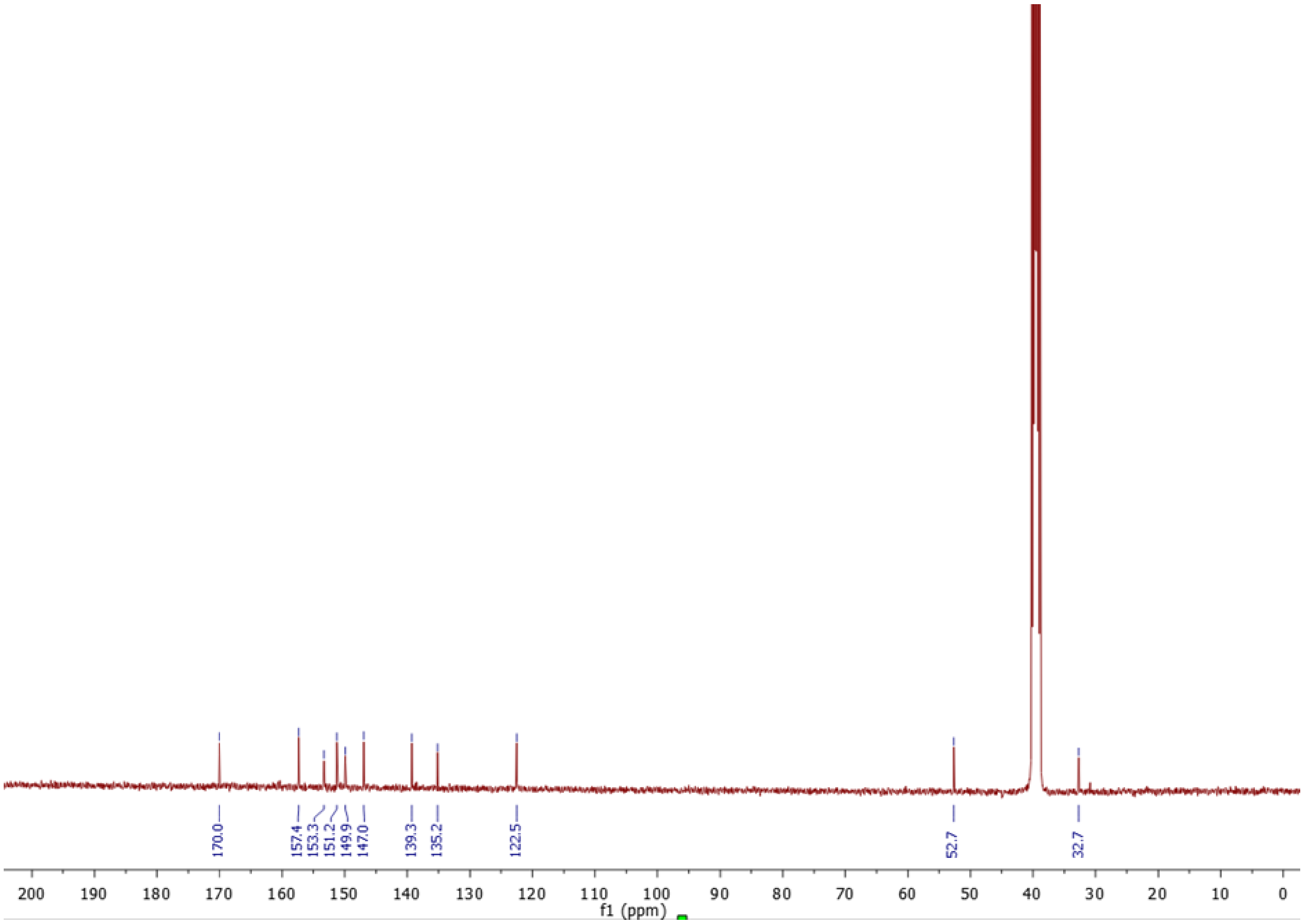

Figure S13. ^{13}C NMR spectrum of **Trz** in DMSO-d_6.

ESI

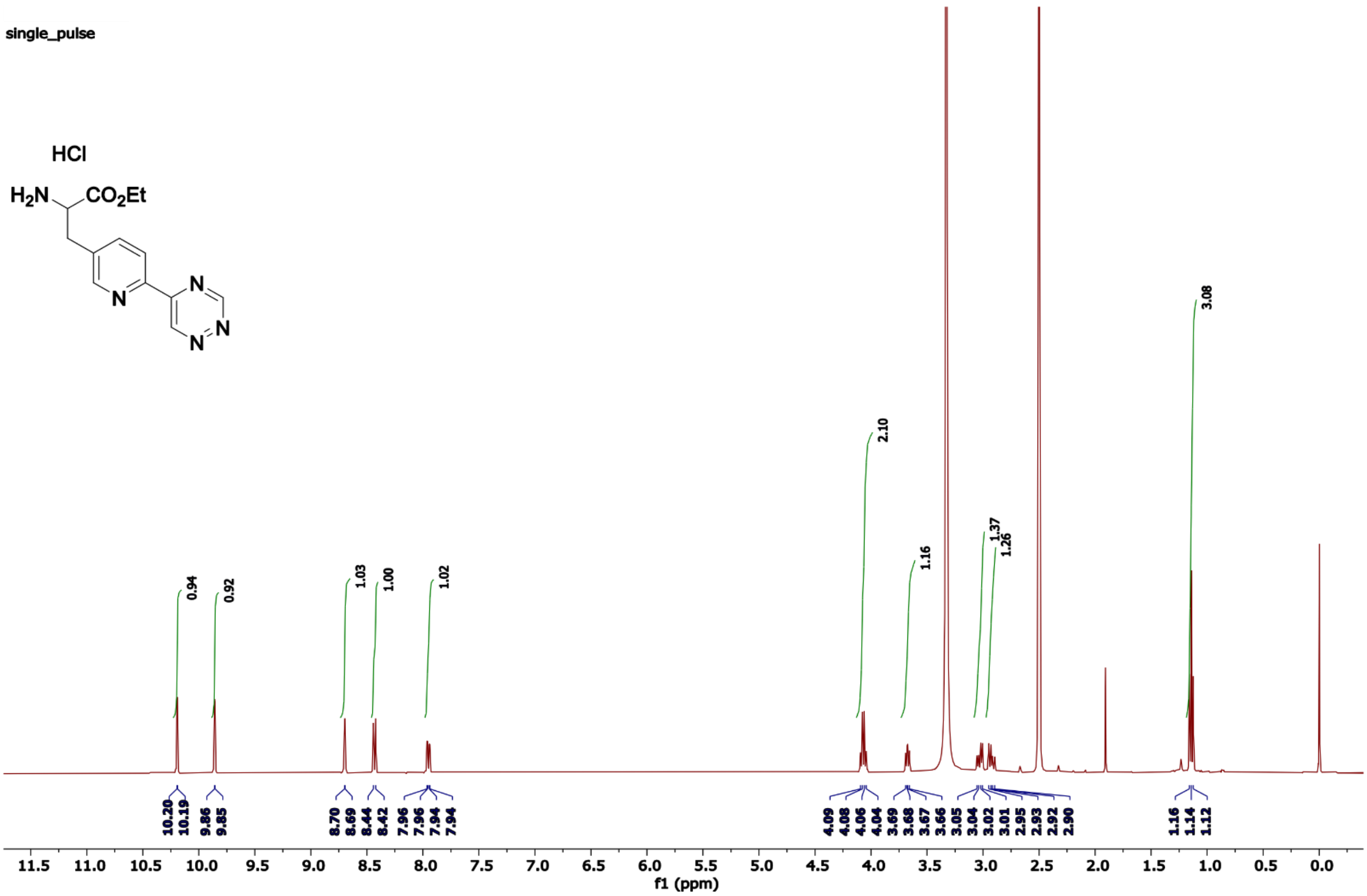

Figure S14. ^{1}H NMR spectrum of Trz-Et in DMSO-d_6.

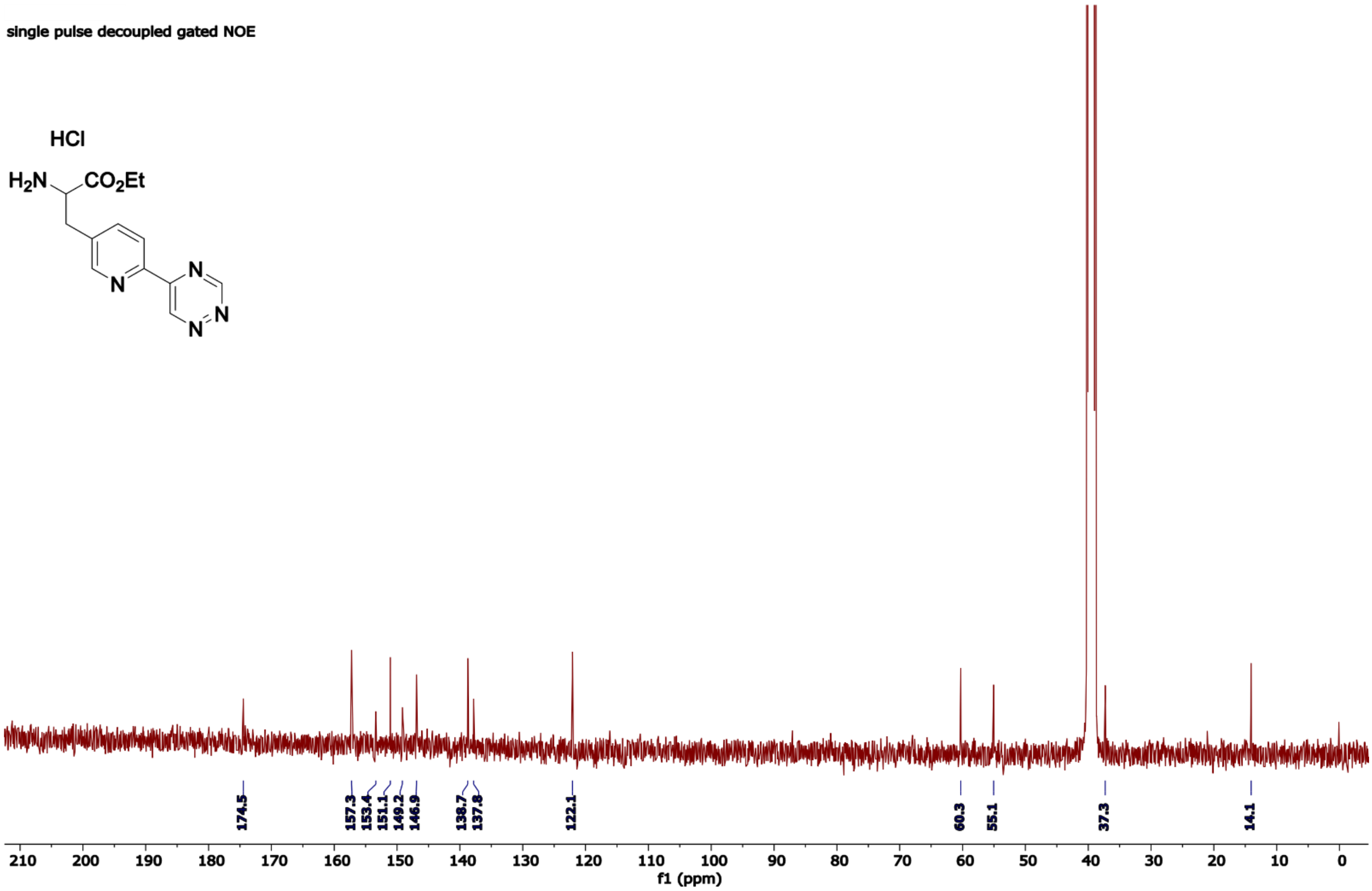

Figure S15. ^{13}C NMR spectrum of Trz-Et in DMSO-d_6.

DEPT with decoupling

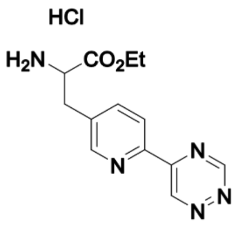

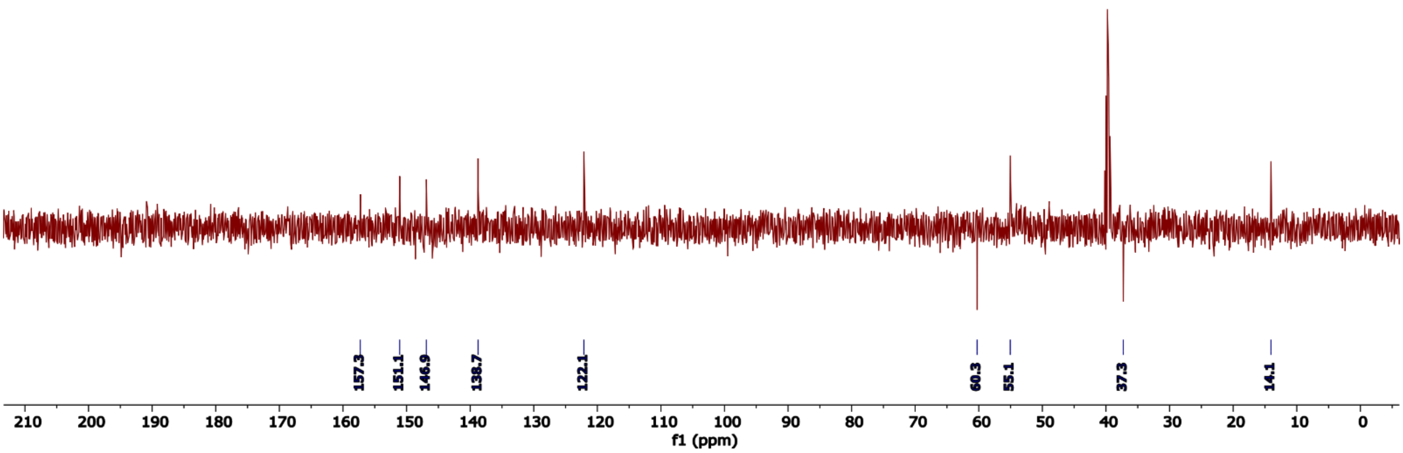

Figure S16. DEPT135 NMR spectrum of Trz-Et in DMSO-d_6.

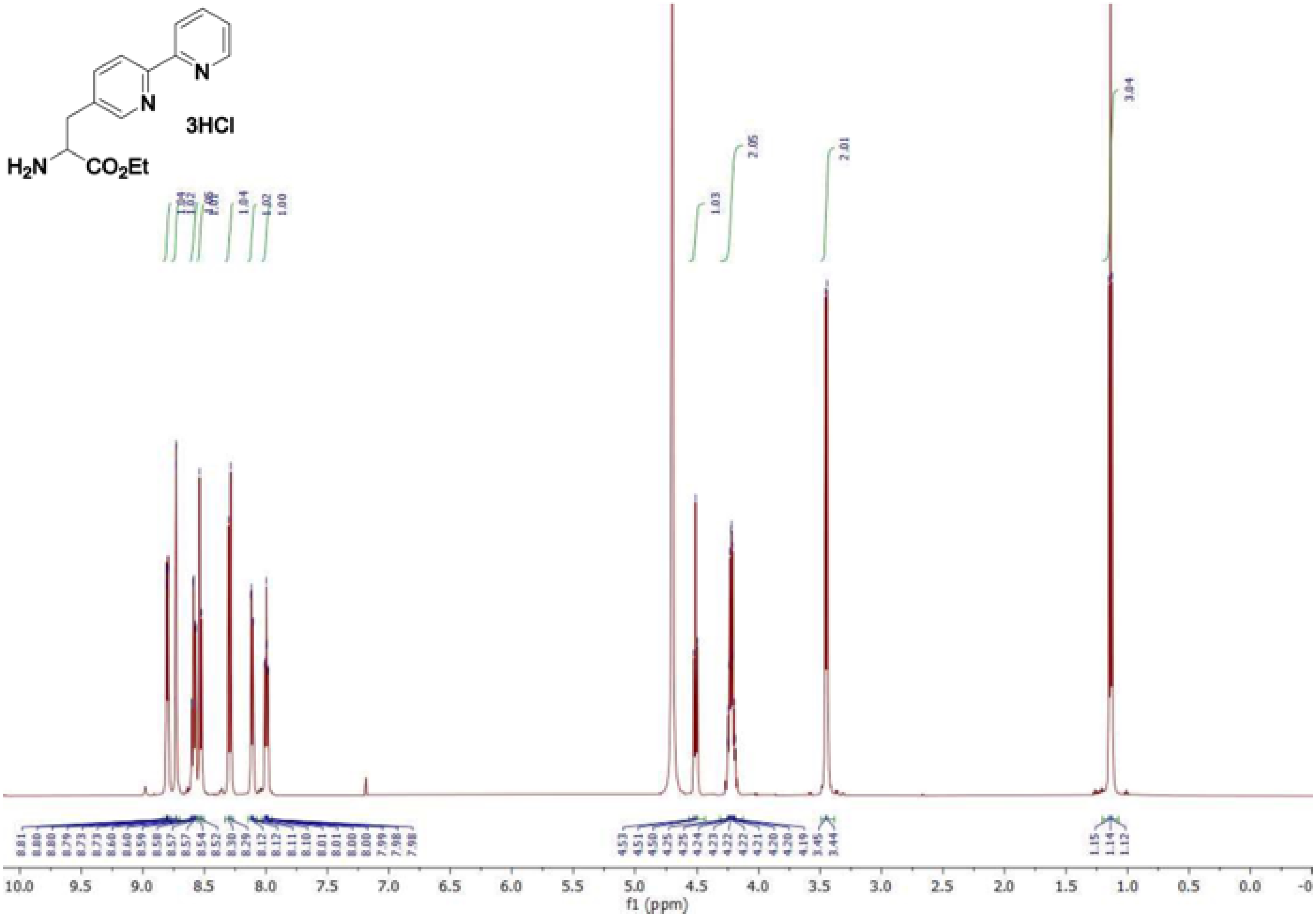

Figure S17. ^{1}H NMR spectrum of BpyAla-Et in D_2O.

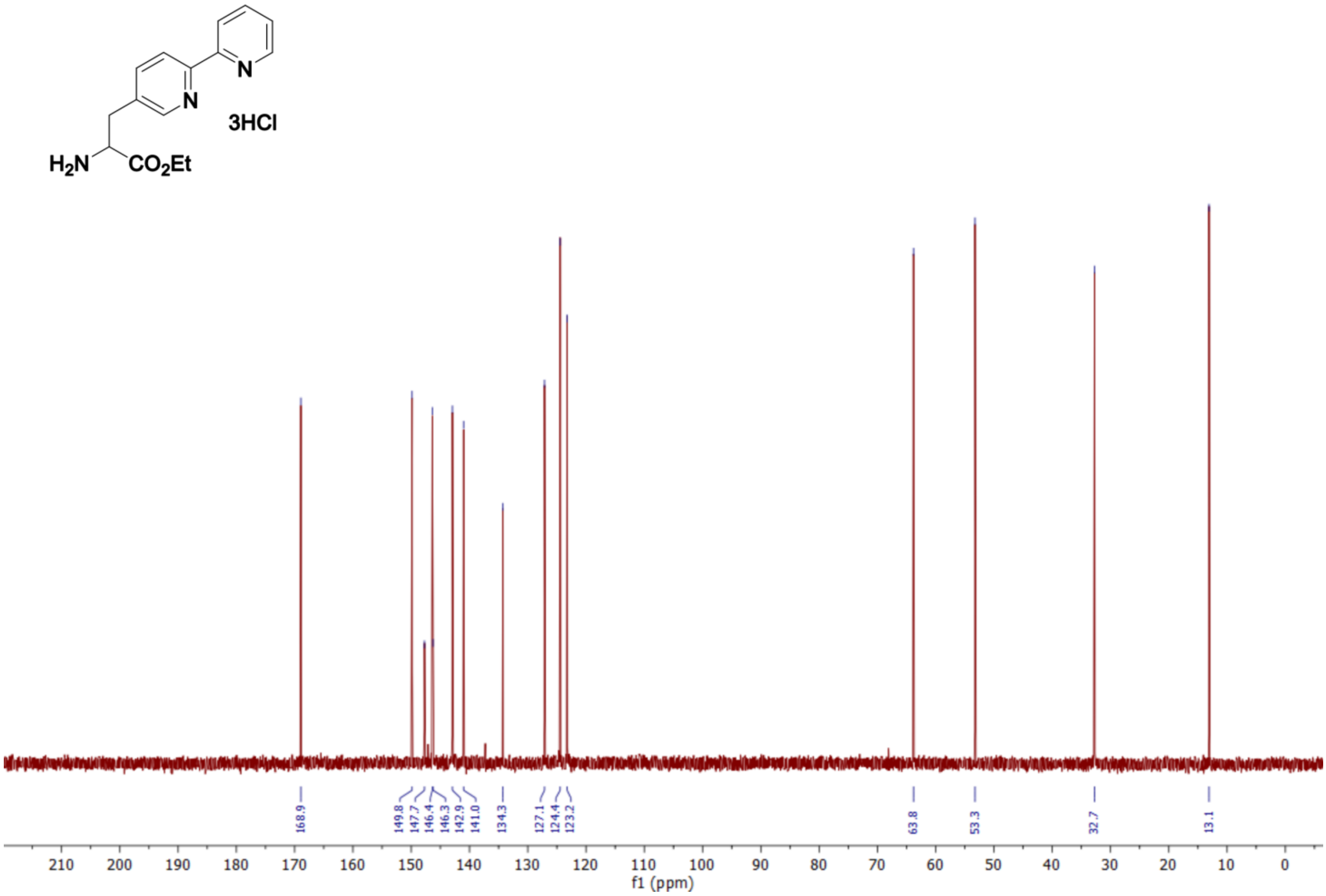

Figure S18. ^{13}C NMR spectrum of BpyAla-Et in D_2O.

Supplementary figures

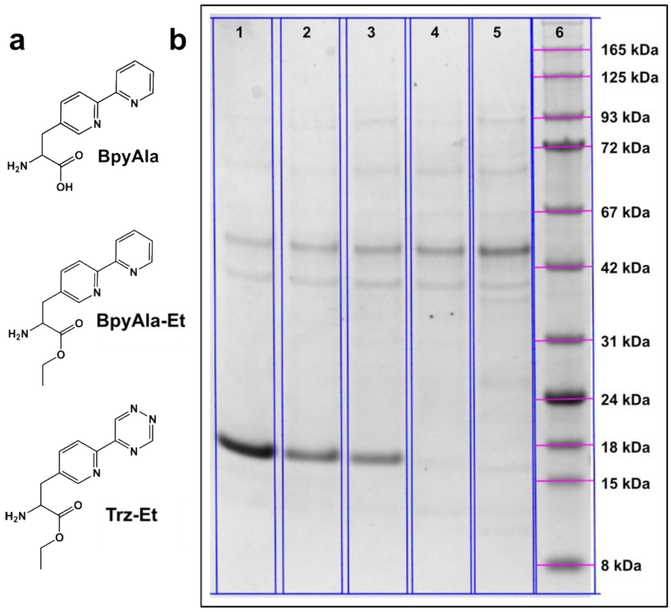

Figure S19. Initial expression test with ethyl ester derivatives of noncanonical amino acids (ncAAs). **a** ncAA bipyridylalanine (BpyAla), ethyl ester derivative BpyAla-Et, and ethyl ester derivative of 1,2,4-triazine ncAA, Trz-Et. **b** SDS–PAGE analysis of expression tests. Lane 1: SCP_BpyAla; lane 2: SCP_BpyAla-Et; SCP_Trz-Et; SCP + DMSO (no ncAA/derivative present); lane 5: uninduced control; and lane 6: protein standard ladder with molecular weights indicated.

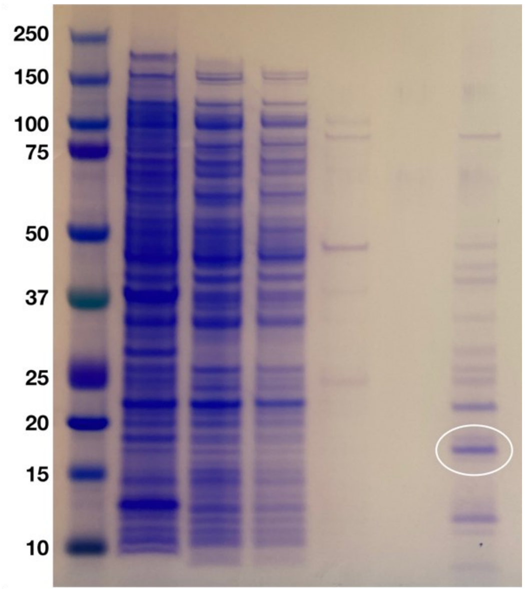

Figure S20. SDS–PAGE gel of SCP-His$_6$_Trz isolated from cell lysate *via* immobilised metal-ion affinity chromatography (IMAC). Samples include: molecular weight standard (1), cell lysate (2), IMAC flow-through (3), IMAC wash fraction (4), IMAC elution fraction (5), dialysed elution fraction (6), and concentrated dialysed fraction (7).

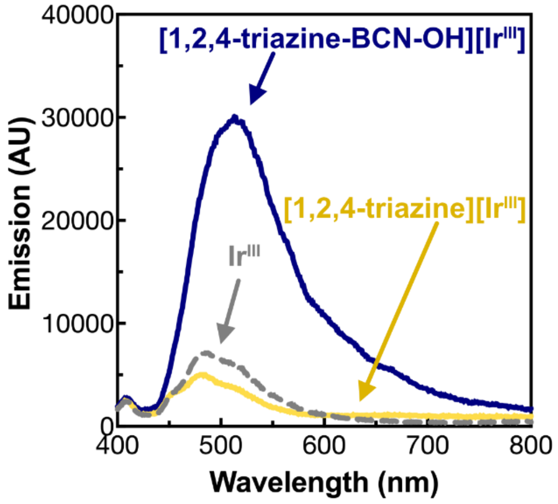

Figure S21. Luminescent emission of the iridium(III) used in this work (Ir^{III}, grey dotted line), complexed with triazine small molecule before (yellow) and after (dark blue) inverse electron-demand Diels–Alder (IEDDA) reaction with BCN-OH, excited at 360 nm. Fluorescence measurements were conducted in 30 mM MES, 5% acetonitrile, pH 6.0, at 25 °C.

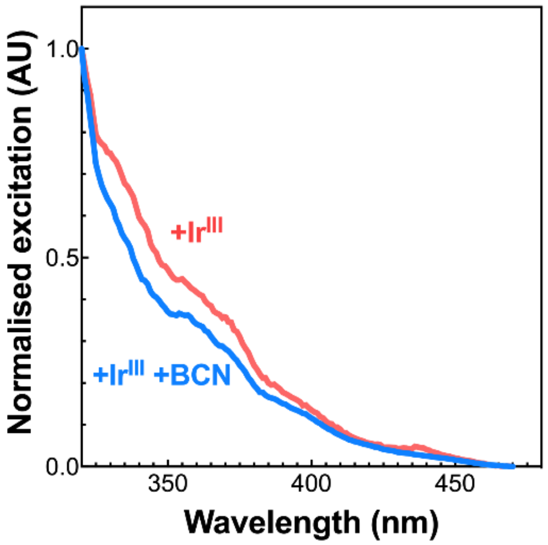

Figure S22. Normalised excitation spectra of [SCP_Trz][Ir^{III}] (pink) and [SCP_Trz]–BCN[Ir^{III}] (blue), at an emission of 520 nm. Fluorescence measurements were conducted in 30 mM MES, 5% acetonitrile, pH 6.0, at 25 °C.

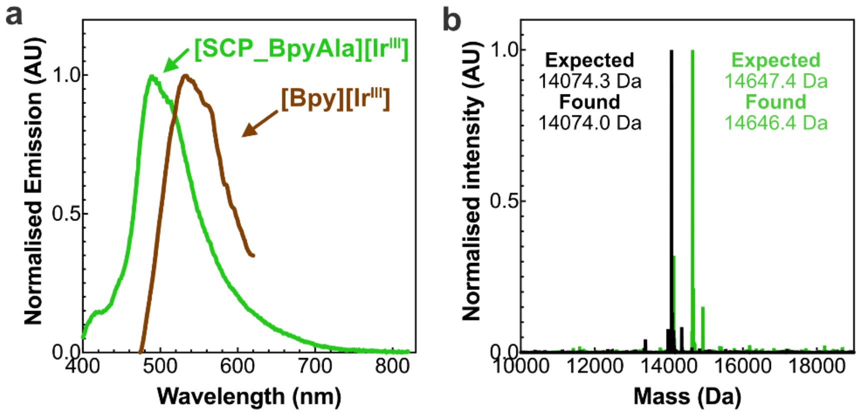

Figure S23: The formation of [SCP_BpyAla][Ir^{III}]. **a** Normalised emission spectra of iridium(III) complexed to the small molecule 2,2'-bipyridine, [Bpy][Ir^{III}] (brown), and SCP exhibiting the non-canonical amino acid 2,2'-bipyridylalanine (BpyAla), to form the complex [SCP_BpyAla][Ir^{III}] (green), excited at 360 nm. Fluorescence measurements were conducted in 30 mM MES, 5% acetonitrile, pH 6.0, at 25 °C. **b** Deconvoluted LC–MS spectra of SCP_BpyAla (black) and [SCP_BpyAla][Ir^{III}].

Protein sequences

Supplementary table 3. SCP sequence before (top) and after (bottom) TEV protease cleavage. Cleavage site underlined. M112 position indicated in bold. SCP* used to indicate the cleaved variant.

**1.** Wild-type SCP-His6 sequence:
MEGGKLQSTFVFEEIGRRLKDIGPEVVKKVNAVFEWHITKGGNIGAKWTIDLKSGSGKVYQGPA<br>KGAADTTIILSDEDFMEVVLGKLDPQKAFFSGRLKARGNIMLSQKLQ**M**ILKDYAKLGSENLYFQG<br>LEHHHHHH<br>DNA:<br>ATGGAGGGTGGCAAGCTGCAAAGCACCTTCGTGTTCGAGGAGATCGGTCGTCGCCTGAAA<br>GACATCGGCCCGGAAGTGGTTAAGAAAGTTAACGCGGTGTTCGAGTGGCACATCACCAAG<br>GGTGGCAACATTGGTGCGAAATGGACCATCGACCTGAAGAGCGGTAGCGGCAAAGTTTAT<br>CAAGGTCCGGCGAAGGGTGCGGCGGATACCACCATCATTCTGAGCGACGAGGATTTTATG<br>GAAGTGGTTCTGGGCAAGCTGGACCCGCAAAAAGCGTTCTTTAGCGGTCGTCTGAAAGCA<br>CGTGGCAACATTATGCTGAGCCAAAAACTGCAAATGATTCTGAAGGATTATGCGAAGCTGG<br>GATCCGAAAACCTGTATTTTCAGGGCCTCGAGCACCACCACCACCACCAC
**2.** *C*-terminal and *N*-terminal cleavable SCP-His6 sequence:
MENLYFQGGGKLQSTFVFEEIGRRLKDIGPEVVKKVNAVFEWHITKGGNIGAKWTIDLKSGSGK<br>VYQGPAKGAADTTIILSDEDFMEVVLGKLDPQKAFFSGRLKARGNIMLSQKLQ**M**ILKDYAKLGS<br>ENLYFQGLEHHHHHH<br>DNA:<br>ATGGAGAACCTGTACTTCCAAGGTGGTGGCAAGCTGCAAAGCACCTTCGTGTTCGAGGAG<br>ATCGGTCGTCGCCTGAAAGACATCGGCCCGGAAGTGGTTAAGAAAGTTAACGCGGTGTTC<br>GAGTGGCACATCACCAAGGGTGGCAACATTGGTGCGAAATGGACCATCGACCTGAAGAGC<br>GGTAGCGGCAAAGTTTATCAAGGTCCGGCGAAGGGTGCGGCGGATACCACCATCATTCTG<br>AGCGACGAGGATTTTATGGAAGTGGTTCTGGGCAAGCTGGACCCGCAAAAAGCGTTCTTTA<br>GCGGTCGTCTGAAAGCACGTGGCAACATTATGCTGAGCCAAAAACTGCAAATGATTCTGAA<br>GGATTATGCGAAGCTGGGATCCGAAAACCTGTATTTTCAGGGCCTCGAGCACCACCACCA<br>CCACCAC
**3.** SCP sequence after double TEV protease cleavage:
GGGKLQSTFVFEEIGRRLKDIGPEVVKKVNAVFEWHITKGGNIGAKWTIDLKSGSGKVYQGPAK<br>GAADTTIILSDEDFMEVVLGKLDPQKAFFSGRLKARGNIMLSQKLQ**M**ILKDYAKLGSENLYFQ

Expected masses

Supplementary table 4. The expected masses of proteins based on their sequences, small molecules, reaction products, and complexes. The expected masses of the protein sequences were determined using Expasy ProtParam webserver.[Supplementary reference 4]

Name	Expected mass (Da)
Sequence 1 (wild-type SCP-His$_6$)	15305.6
Sequence 2 (cleavable SCP-His$_6$)	16028.4
Sequence 3 (cleaved SCP-His$_6$)	13980.2
Methionine 112 (M112)	149.2
Trz	246.1
BpyAla	243.3
BCN-OH	150.2
Dinitrogen	28.0
Iridium(III)	573.1
SCP_Trz-His$_6$ (sequence 1, M112 replaced by Trz)	15402.5
SCP_Trz-His$_6$–BCN-OH (SCP_Trz-His$_6$ + BCN-OH − dinitrogen)	15524.7
SCP_BpyAla (sequence 3, M112 replaced by BpyAla	14074.3
[SCP_BpyAla][Ir^{III}]	14647.4

Supplememtary references

(1) Xie, J.; Liu, W.; Schultz, P. G. A Genetically Encoded Bidentate, Metal-Binding Amino Acid. *Angew. Chem. Int. Ed.* **2007**, *46* (48), 9239–9242. https://doi.org/10.1002/anie.200703397.

(2) Klemencic, E.; Brewster, R. C.; Ali, H. S.; Richardson, J. M.; Jarvis, A. G. Using BpyAla to Generate Copper Artificial Metalloenzymes: A Catalytic and Structural Study. *Catal. Sci. Technol.* **2024**. *14*, 1622-1632. https://doi.org/10.1039/D3CY01648J.

(3) Kozhevnikov, V. N.; Deary, M. E.; Mantso, T.; Panayiotidis, M. I.; Sims, M. T. Iridium(Iii) Complexes of 1,2,4-Triazines as Potential Bioorthogonal Reagents: Metal Coordination Facilitates Luminogenic Reaction with Strained Cyclooctynes. *Chem. Commun.* **2019**, *55* (95), 14283–14286. https://doi.org/10.1039/c9cc06828g.

(4) Gasteiger, E.; Hoogland, C.; Gattiker, A.; Duvaud, S.; Wilkins, M. R.; Appel, R. D.; Bairoch, A. Protein Identification and Analysis Tools on the Expasy Server. *(In) John M. Walker (ed): The Proteomics Protocols Handbook, Humana Press* **2005**, 571–607.
